# The Fabrication and Property Characterization of a Ho_2_YSbO_7_/Bi_2_MoO_6_ Heterojunction Photocatalyst and the Application of the Photodegradation of Diuron under Visible Light Irradiation

**DOI:** 10.3390/ijms25084418

**Published:** 2024-04-17

**Authors:** Liang Hao, Jingfei Luan

**Affiliations:** 1 School of Physics, Changchun Normal University, Changchun 130032, China; hliang0725@163.com; 2 State Key Laboratory of Pollution Control and Resource Reuse, School of the Environment, Nanjing University, Nanjing 210093, China

**Keywords:** Ho_2_YSbO_7_, Ho_2_YSbO_7_/Bi_2_MoO_6_ heterojunction photocatalyst, diuron, visible light exposure, photocatalytic activity, degradation pathway, degradation mechanism

## Abstract

A novel photocatalytic nanomaterial, Ho_2_YSbO_7_, was successfully synthesized for the first time using the solvothermal synthesis technique. In addition, a Ho_2_YSbO_7_/Bi_2_MoO_6_ heterojunction photocatalyst (HBHP) was prepared via the hydrothermal fabrication technique. Extensive characterizations of the synthesized samples were conducted using various instruments, such as an X-ray diffractometer, a Fourier transform infrared spectrometer, a Raman spectrometer, a UV-visible spectrophotometer, an X-ray photoelectron spectrometer, and a transmission electron microscope, as well as X-ray energy dispersive spectroscopy, photoluminescence spectroscopy, a photocurrent test, electrochemical impedance spectroscopy, ultraviolet photoelectron spectroscopy, and electron paramagnetic resonance. The photocatalytic activity of the HBHP was evaluated for the degradation of diuron (DRN) and the mineralization of total organic carbon (TOC) under visible light exposure for 152 min. Remarkable removal efficiencies were achieved, with 99.78% for DRN and 97.19% for TOC. Comparative analysis demonstrated that the HBHP exhibited markedly higher removal efficiencies for DRN compared to Ho_2_YSbO_7_, Bi_2_MoO_6_, or N-doped TiO_2_ photocatalyst, with removal efficiencies 1.13 times, 1.21 times, or 2.95 times higher, respectively. Similarly, the HBHP demonstrated significantly higher removal efficiencies for TOC compared to Ho_2_YSbO_7_, Bi_2_MoO_6_, or N-doped TiO_2_ photocatalyst, with removal efficiencies 1.17 times, 1.25 times, or 3.39 times higher, respectively. Furthermore, the HBHP demonstrated excellent stability and reusability. The mechanisms which could enhance the photocatalytic activity remarkably and the involvement of the major active species were comprehensively discussed, with superoxide radicals identified as the primary active species, followed by hydroxyl radicals and holes. The results of this study contribute to the advancement of efficient heterostructural materials and offer valuable insights into the development of sustainable remediation strategies for addressing DRN contamination.

## 1. Introduction

To address the pressing issue of food scarcity resulting from population growth and limited agricultural resources, the use of pesticides has become indispensable in enhancing crop yield [1,2,3]. Among various pesticides, diuron (DRN) stands out as a widely utilized herbicide due to its universality and effectiveness at weed control [4,5,6]. However, the residual presence of DRN in agricultural production poses significant risks to organisms due to its carcinogenic and genotoxic properties [7,8]. Recognizing its potential harm, the European Union’s Water Framework Directive designated DRN as a hazardous substance, and the United States Environmental Protection Agency classified it as a “known probable” human carcinogen [9]. In order to mitigate the negative effects of DRN on water resources and organisms, it is imperative to degrade DRN in pesticide wastewater into mineral or relatively stable innoxious organic solids.

The traditional process of water pollution treatment involved the fusion of physical, chemical, and biological methods [10,11,12]. Nonetheless, with the advancement of industrial processes, effluents have become increasingly complex, making it more challenging to completely remove organic pollutants using these conventional methods. Moreover, these conventional methods suffer from issues such as incomplete degradation, low efficiency, high cost, and the potential for secondary pollution risks [13,14,15]. Thus, additional treatment steps should be incorporated into effluent treatment projects to ensure the further removal of toxic materials.

Semiconductor photocatalysis technology, recognized as an advanced oxidation process (AOP), has emerged as a highly promising and effective approach for water pollution treatment [16,17,18,19]. Its notable advantages include low cost, high efficiency, absence of secondary pollution, recyclability, and environmentally friendly characteristics [20,21,22]. By harnessing solar energy, semiconductor photocatalysis successfully degraded highly stable organic molecules, addressing the persistent challenge of water pollution. During the photocatalytic process, semiconductor photocatalysts are excited by solar radiation, resulting in the generation of electron–hole pairs. These pairs further produce reactive oxygen species (ROS) such as hydroxyl and superoxide radicals, which facilitate the oxidation process of pollutants, ultimately mineralizing them into innocuous CO_2_ and H_2_O [23,24,25]. While ultraviolet light constitutes only 5% of solar radiation, visible light represents a larger portion, accounting for 45% of solar energy. However, traditional semiconductor photocatalysts such as titanium dioxide and zinc oxide require ultraviolet or near-ultraviolet radiation for activation, resulting in a significant waste of energy [26,27,28,29,30]. Hence, synthesizing composite photocatalytic materials is crucial for effectively utilizing solar energy as composite catalysts have shown significant improvements in terms of sunlight absorption range and photocatalytic capability compared to single metal oxide catalysts [31,32]. Previous studies have demonstrated the favorable photocatalytic capability of A_2_B_2_O_7_ and AB_2_O_6_ mixtures under visible light exposure (VLE) [33,34,35,36,37,38,39]. For instance, Devi et al. achieved a photocatalytic removal efficiency (REF) of 66% for methylene blue (0.1 mmol/L) using the photocatalyst YGdTi_2_O_7_ under VLE for 180 min [40]. Similarly, Zhang et al. reported an REF of 69.7% for methyl orange (20 mg/L) using the photocatalyst Gd_2_Ce_2_O_7_ powder under VLE for 300 min [41]. Furthermore, Bi_2_MoO_6_, an important Aurivillius-phase perovskite, has garnered great attention for its excellent photocatalytic activity under VLE [42,43]. Zhao et al. found that Bi_2_MoO_6_ powder achieved an REF of 65.6% for methyl blue (20 mg/L) under VLE for 240 min, while Li et al. demonstrated a photocatalytic REF of 60% for rhodamine B (10 mg/L) using Bi_2_MoO_6_ under VLE for 120 min [44,45]. Building upon these promising findings, we have integrated Bi_2_MoO_6_ into our latest research to develop a novel photocatalytic material that exhibited enhanced performance for water pollution treatment. Further details will be elaborated in the subsequent sections.

In our previous research, we extensively explored the potential for structure tuning in the stable pyrochlore-structured photocatalyst Bi_2_InTaO_7_ [46]. The groundbreaking research conducted by Barrocas et al. has demonstrated remarkable photocatalytic degradation efficiencies for methylene blue dye pollutants using Bi_2_S_3_–ZnS/TNF nanocomposite photocatalytic materials, as well as for Rhodamine 6G dye pollutants using Ca_1−x_Ln_x_MnO_3_ and Ca_0_._6_Ho_0_._4_MnO_3_ film photocatalytic materials [47,48,49]. These remarkable findings have served as a significant source of inspiration for our own research. Moreover, the advancements made by Cai et al. in the degradation of methyl orange dye utilizing Ho-doped TiO_2_ photocatalysts have also been highly beneficial to our work [50]. Expanding on the above analysis, we hypothesized that the carrier concentration could be enhanced through specific element substitutions. Experimental results confirmed the advanced photocatalytic potential of the novel photocatalyst Ho_2_YSbO_7_.

To further enhance the efficacy of photocatalysts, various methods have been explored, including morphological control, band gap engineering, and the construction of heterojunction structures [51,52,53,54]. Among these techniques, the construction of heterojunction photocatalysts has garnered significant attention [55,56]. The formation of a strong internal electric field within heterojunction photocatalytic materials effectively separated and transferred photoinduced carriers (PICs), thereby reducing their recombination reactions [57,58]. This promoted the generation of ROS, ultimately reinforcing the photocatalytic capability of the catalyst [16]. In recent years, substantial progress has been made in the field of semiconductor heterojunction photocatalysts. For instance, Luo et al. reported on the construction of a SnS_2_/Ag_3_PO_4_ heterojunction photocatalyst, which exhibited enhanced degradation performance compared to individual SnS_2_ and Ag_3_PO_4_ catalysts [59]. Similarly, Zhang et al. demonstrated an improved photodegradation rate of tetracycline hydrochloride using a g-C_3_N_4_/TiO_2_ heterojunction photocatalyst compared to each standalone catalyst [60].

Motivated by these fundamental insights, we have developed a novel heterostructure photocatalytic material, Ho_2_YSbO_7_/Bi_2_MoO_6_, for the effective removal of DRN from pesticide wastewater under VLE. Experimental results confirmed the exceptional performance of the Ho_2_YSbO_7_/Bi_2_MoO_6_ heterojunction photocatalyst (HBHP), achieving a remarkable DRN (0.032 mmol/L) REF of 99.78% within 152 min of VLE. The remarkable superiority of HBHP was evident in its exceptional performance compared to conventional TiO_2_. Within the same irradiation time, TiO_2_ achieved a removal efficiency of less than 40% for a DRN initial concentration of 0.0043 mmol/L under UV light condition [61]. Similarly, the nickel organic xerogel achieved a removal efficiency of less than 80% for a DRN initial concentration of 0.021 mmol/L under simulated sunlight conditions [62]. These striking disparities clearly demonstrate the significantly enhanced effectiveness of the tested photocatalyst in degrading DRN. Additionally, the photocatalytic degradation efficiency of DRN in pesticide wastewater under VLE was evaluated for various photocatalysts, including HBHP, Ho_2_YSbO_7_, Bi_2_MoO_6_, and nitrogen-doped titanium dioxide. This study presents a significant innovation by successfully synthesizing the novel visible-light-driven Ho_2_YSbO_7_ photocatalytic material with excellent photocatalytic properties using the solvothermal synthesis technique. These findings represent a remarkable advancement in the development of efficient and environmentally friendly photocatalytic systems for DRN degradation in pesticide wastewater.

## 2. Result and Discussion

### 2.1. X-ray Diffraction Analysis

**Figure 1:**
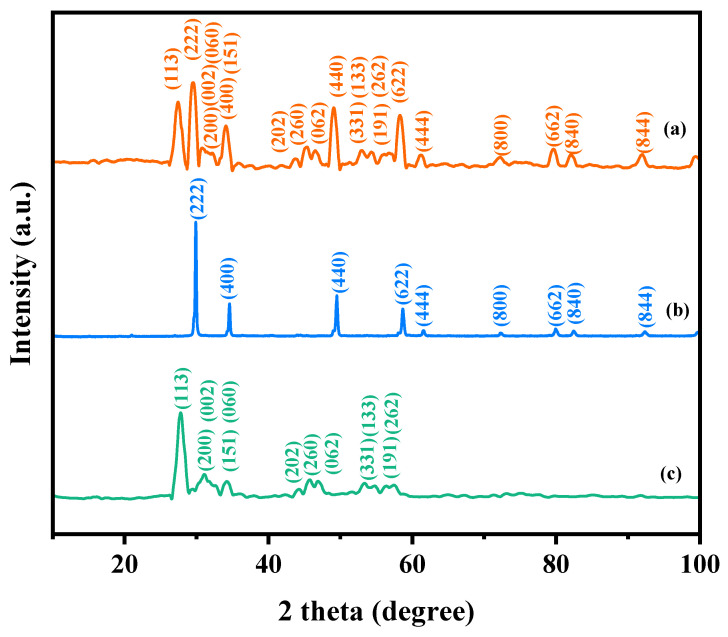
XRD imageries: (**a**) HBHP, (**b**) Ho_2_YSbO_7_, and (**c**) Bi_2_MoO_6_.

**Figure 2:**
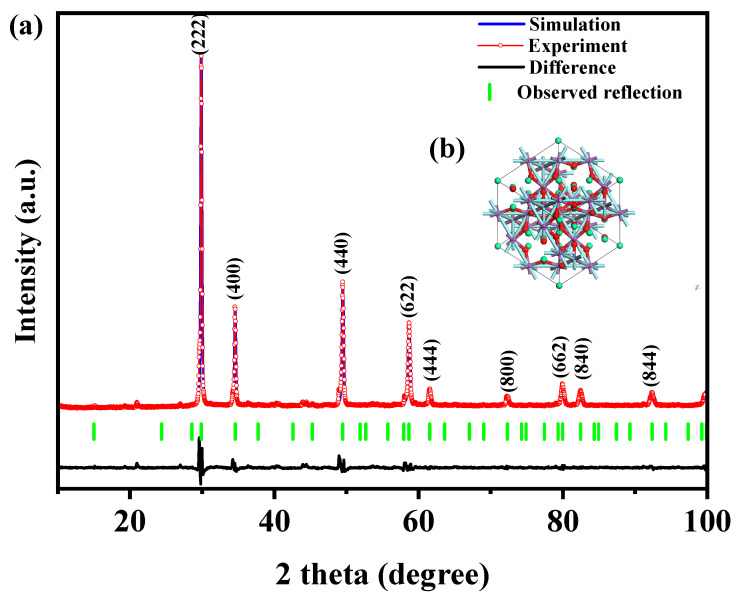
(**a**) XRD imagery and Rietveld refinement and (**b**) the atomic structure (red atom: O; cyan atom: Y or Sb; green atom: Ho) of Ho_2_YSbO_7_.

**Figure 3:**
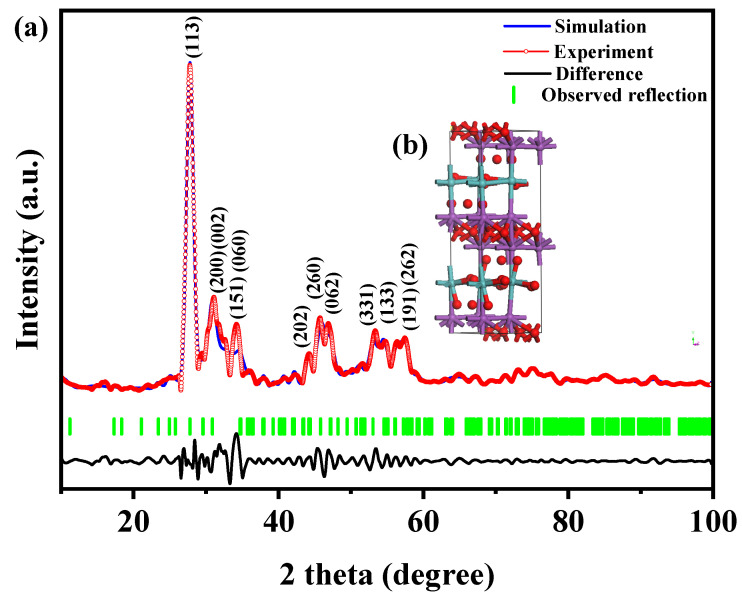
(**a**) XRD pattern and Rietveld refinement and (**b**) the atomic structure (Red atom: O; blue atom: Mo; purple atom: Bi) of Bi_2_MoO_6_.

Figure 1 presents the XRD patterns of the HBHP, Ho_2_YSbO_7_, and Bi_2_MoO_6_. The observed peaks and crystal planes in the XRD pattern of the HBHP match those of Ho_2_YSbO_7_ and Bi_2_MoO_6_, affirming the successful synthesis of the HBHP [63,64,65]. To verify the crystal structures, the XRD data of Ho_2_YSbO_7_ and Bi_2_MoO_6_ were further subjected to Rietveld refinement using the Materials Studio program, as shown in Figure 2a and Figure 3a, respectively. The refined results for Ho_2_YSbO_7_ (R_P_ = 5.45%) and Bi_2_MoO_6_ (R_P_ = 6.29%) exhibit exceptional agreement between the experimental and theoretical intensities. This confirms the pyrochlore-type structure of Ho_2_YSbO_7_ and the structural arrangement of Bi_2_MoO_6_, consisting of (MoO_4_)^2−^ perovskite layers organized between (Bi_2_O_2_)^2+^ layers [42,66]. Moreover, both compounds exhibit single-phase behavior, with Ho_2_YSbO_7_ crystallizing in the cubic crystal system with the space group Fd3m and Bi_2_MoO_6_ adopting the orthorhombic crystal system with the space group Pca21. The lattice constant of Ho_2_YSbO_7_ was determined to be 10.355 Å, and for Bi_2_MoO_6_, the lattice constants were found to be a = 5.432 Å, b = 16.370 Å, and c = 5.447 Å. The refinement model, which accounted for the presence of oxygen atoms, demonstrates excellent agreement between the experimental and theoretical intensities. Additionally, the XRD pattern of N-doped TiO_2_ (NTO) is depicted in Figure S1, showing the characteristic lattice planes of the anatase phase of TiO_2_ (JCPDS No. 21-1272) [67,68,69].

Figure 2b and Figure 3b illustrate the atomic structures of Ho_2_YSbO_7_ and Bi_2_MoO_6_, respectively. These crystal structures were constructed by us using the corresponding space group, crystal system, lattice constants, atomic coordinates, and structural parameters. Furthermore, Table 1 and Table 2 present the atomic coordinates and structural parameters for Ho_2_YSbO_7_ and Bi_2_MoO_6_, respectively [70,71]. These detailed analyses provide strong evidence for the structural stability of the synthesized compounds and underscore their potential as highly efficient photocatalysts in various applications.

The observed distortions in the MO_6_ octahedra (M = Y^3+^ and Sb^5+^) of Ho_2_YSbO_7_ indicate a crystal structure distortion, which has been proven to enhance photocatalytic efficiency in previous studies [46]. Additionally, the unique crystal structure of Ho_2_YSbO_7_, composed of interconnected MO_6_ (M = Y^3+^ and Sb^5+^) octahedra through Ho^3+^ ions, exhibits distinctive Ho-O bond distances and bond angles. In the crystal structure of Ho_2_YSbO_7_, there are two types of Ho–O bond lengths: the six longer Ho–O(1) bond lengths (4.766 Å) and the two shorter Ho–O(2) bond lengths (2.242 Å). The six M–O(1) (M = Y^3+^ and Sb^5+^) bond lengths were determined as 1.988 Å, and the M–Ho (M = Y^3+^ and Sb^5+^) bond length was 3.661 Å. The M–O–M (M = Y^3+^ and Sb^5+^) bond angles were measured as 134.020°, while the Ho-M-Ho (M = Y^3+^ and Sb^5+^) bond angles were 135.00° in the crystal structure of Ho_2_YSbO_7_. The Ho–M–O (M = Y^3+^ and Sb^5+^) bond angles were found to be 137.431°. The M–O–M bond angles in Ho_2_YSbO_7_ could impact the mobility of PICs, thus influencing their ability to reach the surface reaction centers and affect the photocatalytic efficiency [46]. Moreover, the larger Ho–Y–O and Ho–Sb–O bond angles in Ho_2_YSbO_7_ may further enhance its photocatalytic properties.

The remarkable photocatalytic performance of Ho_2_YsbO_7_ could be attributed to its unique crystalline structure and electronic properties. The insights gained into the crystallographic and electronic characteristics of Ho_2_YsbO_7_ are crucial for understanding and optimizing its photocatalytic capabilities.

### 2.2. FTIR Analysis

**Figure 4:**
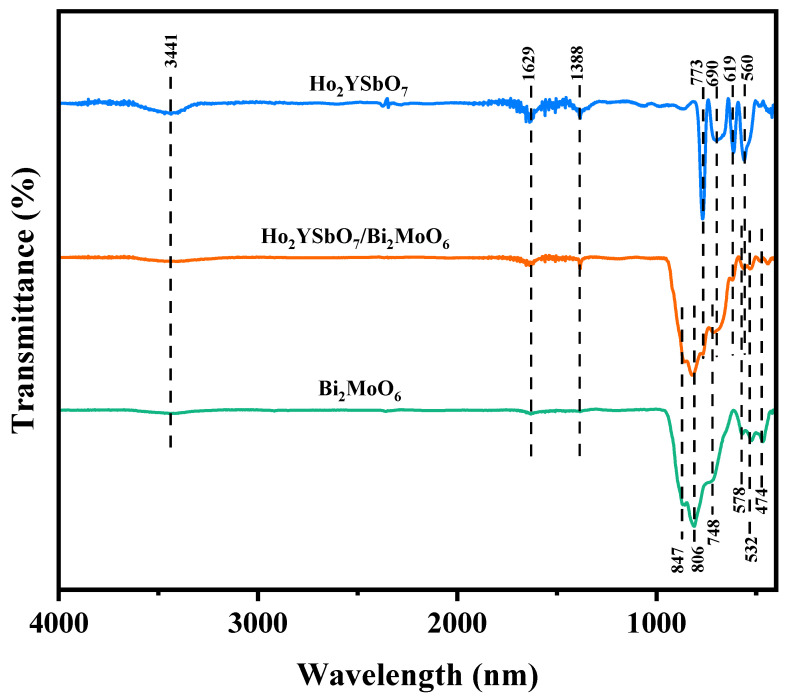
FTIR spectra of Ho_2_YsbO_7_, Bi_2_MoO_6_, and HBHP.

To investigate the presence of functional groups and chemical bonds in HBHP, Ho_2_YsbO_7_, and Bi_2_MoO_6_, FTIR spectra were collected using an FTIR spectrometer, as shown in Figure 4. The bending vibrations of Ho-O were observed at 560 cm^−1^ [72,73]. The bending vibrations of Sb–O and Sb–O–Sb were detected at 619 cm^−1^ and 690 cm^−1^, respectively [74,75]. The stretching vibrations of Y-O appeared at 713 cm^−1^ [76]. The bands observed at 847 cm^−1^ and 806 cm^−1^ could be assigned to the asymmetric and symmetric stretching modes of MoO_6_, respectively, involving vibrations of the apical oxygen atoms [77]. The 748 cm^−1^ mode represented the asymmetric stretching mode of MoO_6_, involving vibrations of the equatorial oxygen atoms. The bands observed at 578 cm^−1^ and 532 cm^−1^ correspond to the bending vibrations of MoO_6_ [77]. Additionally, a small band at 474 cm^−1^ could be attributed to the stretching and bending vibrations of BiO_6_ octahedra [77].

The broad bands centered around 3441 cm^−1^ were indicative of the stretching vibration mode of hydroxyl (OH) groups adsorbed with water [78]. Similarly, the absorption bands observed at 1629 cm^−1^ correspond to the bending vibration of surface OH groups [78]. Additionally, the peaks observed at 1388 cm^−1^ could be attributed to the vibrational mode of C-H bonds [78].

### 2.3. Raman Analysis

**Figure 5:**
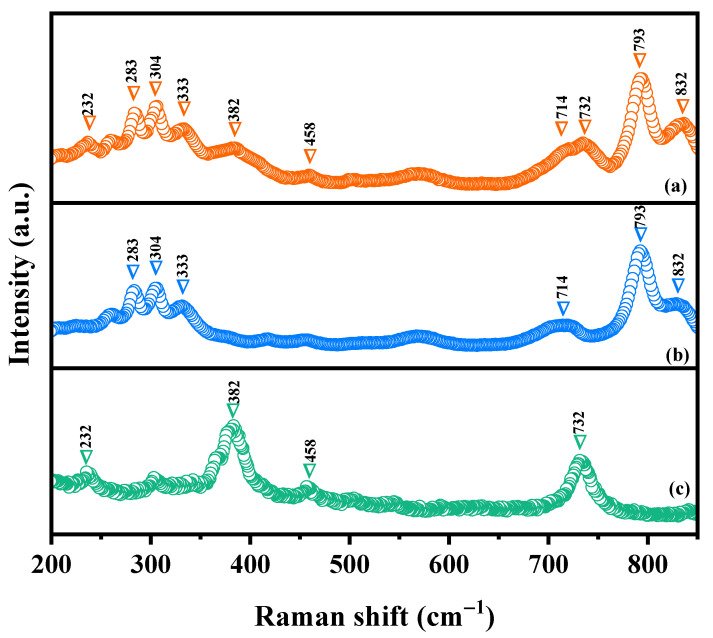
Raman spectra of (**a**) HBHP, (**b**) Bi_2_MoO_6_, and (**c**) Ho_2_YSbO_7_.

Raman spectroscopy was deployed to investigate the chemical bonding characteristics as well as the molecular vibrations and rotations of HBHP, Ho_2_YSbO_7_, and Bi_2_MoO_6_ in this study. Figure 5 illustrates the obtained Raman spectra for Ho_2_YSbO_7_, Bi_2_MoO_6_, and HBHP. The Raman spectra of Ho_2_YSbO_7_ exhibited several notable modes, such as the A_1_-type stretching vibration of the Sb–O–Sb bond at 232 cm^−1^ and the A_g_-type stretching vibration of the Ho–O bond at 382 cm^−1^ [79,80]. Additionally, the Y–O bond displayed an A_g_ bending vibration mode at 458 cm^−1^ [81]. The presence of another peak at 710 cm^−1^ could be attributed to the Sb–O–Sb stretching with T_2_ symmetry [79]. In case of Bi_2_MoO_6_, strong Raman modes observed at 283 cm^−1^ were associated with the E_g_ bending vibrations. The modes at 304 cm^−1^ and 333 cm^−1^ were identified as the E_u_ symmetric bending vibrations [77]. Moreover, the 714 cm^−1^ mode represented the asymmetric stretching vibration (E_u_ mode) of the MoO_6_ octahedra, involving the movement of equatorial oxygen atoms connecting the MoO_6_ octahedra within the layers [77]. The Raman vibrations at 793 cm^−1^ and 832 cm^−1^ were, respectively, attributed to the A_1g_ symmetric and A_2u_ asymmetric stretching vibrations of the MoO_6_ octahedra, involving the motion of apical oxygen atoms directed towards the (Bi_2_O_2_)^2+^ layers [77,82,83]. These observed peaks confirm the presence of pure phases, consistent with the XRD results. The distinctive peaks in the Raman spectrum of HBHP at 232 cm^−1^, 283 cm^−1^, 304 cm^−1^, 333 cm^−1^, 382 cm^−1^, 458 cm^−1^, 714 cm^−1^, 732 cm^−1^, 793 cm^−1^, and 832 cm^−1^ validate the unique characteristics of both Ho_2_YSbO_7_ and Bi_2_MoO_6_. These findings further support the successful integration of both materials within the heterostructure photocatalyst.

### 2.4. UV-Vis Diffuse Reflectance Spectra

To investigate the band structure of the synthesized samples, a detailed analysis of the absorption spectra of Ho_2_YSbO_7_, Bi_2_MoO_6_, and HBHP was conducted, as presented in Figure S2a. The absorption verges of the diffuse reflectance spectra of Ho_2_YSbO_7_ and Bi_2_MoO_6_ were observed at around 450 nm and 505 nm, respectively. Notably, HBHP exhibited a distinctive absorption verge at approximately 520 nm, demonstrating a significant red-shift compared to Ho_2_YSbO_7_ and Bi_2_MoO_6_. This observation suggests that HBHP possesses a higher light absorption capacity than either Ho_2_YSbO_7_ or Bi_2_MoO_6_.

To determine the energy difference between the bands in these samples, the Kubelka–Munk function (1) was employed [84,85]. This method involves identifying the cross-section point at which the photon energy (*hν*) axis intersects with the line extrapolated from the linear section of the absorption spectrum peaks. By utilizing this approach, the energy disparity between the bands in the samples could be assessed.

(1) $$\frac{{\left[1-R_{d}(h\nu )\right]}^{2}}{2R_{d}(h\nu )}=\frac{\alpha (h\nu )}{S}$$

In this given equation, the scattering index, the diffuse reflection, and the absorbance factor of radiation were assigned to *S*, *R_d_*, and *α*, respectively.

The optical absorption characteristics close to the band verges of the samples cohered with the Equation (2) [86,87]:

(2) (αhν)1n=A(hν−Eg)

The given equation introduced in this study involves the symbols *A*, *α*, *E_g_*, and *ν*, which, respectively, represent the relative factor, absorbance factor, band energy difference, and photon frequency. The parameter *n* is utilized to characterize the transition property of photoinduced electrons, with a value of 1/2 for direct transition and a value of 2 for indirect transition [88,89].

Analyzing the results presented in Figure S2b, the calculated band gap values for Ho_2_YSbO_7_ and Bi_2_MoO_6_ were determined as 2.686 eV and 2.483 eV, respectively. Both materials exhibited an *n* value close to 2, indicating indirect transition. Similarly, the band gap value for HBHP was computed as 2.387 eV, suggesting indirect transition. The significantly lower *E_g_* value of HBHP compared to those of Ho_2_YSbO_7_ and Bi_2_MoO_6_ provides strong evidence of HBHP’s superior light absorption capacity.

Similarly, Figure S3a presents the absorption spectra of NTO and pristine TiO_2_. In the diffuse reflectance spectra of TiO_2_, the absorption edge was observed at approximately 400 nm. Notably, NTO exhibited a distinct absorption edge at around 445 nm, indicating a significant red-shift compared to TiO_2_. Moreover, Figure S3b enables the determination of the band gap values for NTO and TiO_2_ as 2.781 eV and 3.064 eV, respectively. The considerably lower band gap value and the higher absorption edge observed in NTO compared to TiO_2_ provide compelling evidence of its superior light absorption capacity within the visible light range, confirming the successful fabrication of NTO [67,68,69].

### 2.5. X-ray Photoelectron Spectroscopy Analysis

X-ray photoelectron spectroscopy (XPS) was utilized to evaluate the chemical makeup and oxidation states of HBHP, Ho_2_YSbO_7_, and Bi_2_MoO_6_. The survey spectrum in Figure S4 indicates the presence of Ho, Y, Sb, Bi, Mo, and O elements in HBHP, with the carbon peak serving as a calibration reference. When comparing the spectrum of Ho_2_YSbO_7_ with HBHP, distinct bismuth and molybdenum signals were observed in the latter, indicating the inclusion of Bi_2_MoO_6_ in HBHP.

**Figure 6:**
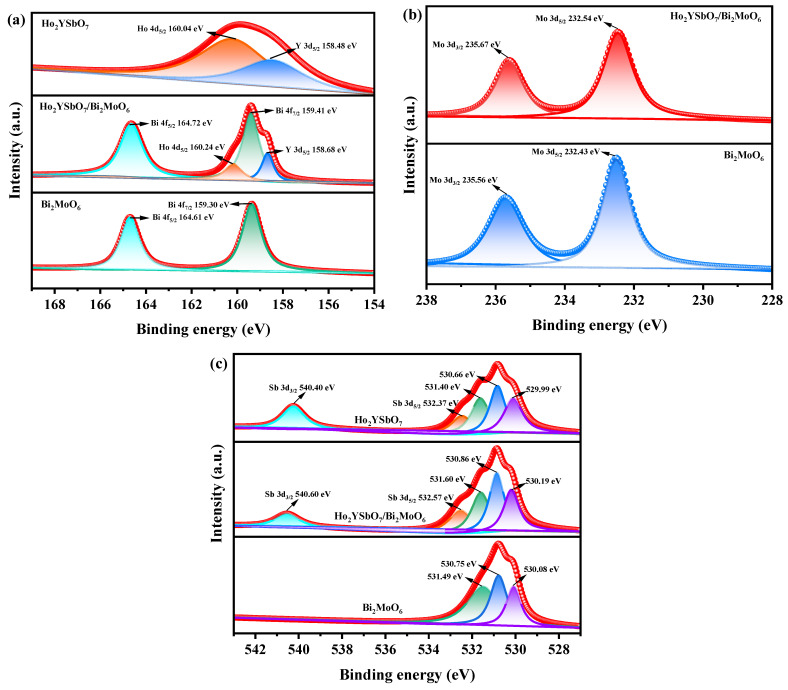
The corresponding high-resolution XPS spectra of (**a**) Ho 4d, Y 3d, and Bi 4f; (**b**) Mo 3d; (**c**) and O 1s and Sb 3d of HBHP, Ho_2_YSbO_7_, and Bi_2_MoO_6_.

Figure 6a,b display the spectral peaks of Ho 4d_5/2_, Y 3d_5/2_, Bi 4f_7/2_, Bi 4f_5/2_, Mo 3d_5/2_, and Mo 3d_3/2_ in Ho_2_YSbO_7_, Bi_2_MoO_6_, and HBHP. These peaks exhibited slight shifts towards higher binding energies in HBHP compared to the former compounds, indicating notable interfacial interactions between Ho_2_YSbO_7_ and Bi_2_MoO_6_ within the heterostructure. The spin–orbit separation values between Bi 4f_7/2_ and Bi 4f_5/2_ were 5.31 eV for both Bi_2_MoO_6_ and HBHP, confirming the exclusive presence of Bi^3+^ [90]. Similarly, the spin–orbit separation values between Mo 3d_3/2_ and Mo 3d_5/2_ were 3.13 eV for both Bi_2_MoO_6_ and HBHP, indicating the exclusive presence of Mo^6+^ [91].

Figure 6c showcases the deconvoluted O 1s spectrum of HBHP, Ho_2_YSbO_7_, and Bi_2_MoO_6_. The observed peaks at 530.19 eV, 529.99 eV, and 530.08 eV correspond to the lattice oxygen [92]. Furthermore, the peaks at 530.86 eV, 530.66 eV, and 530.75 eV signify the signal originating from hydroxyl groups [93]. Additionally, the peaks at 531.60 eV, 531.40 eV, and 531.49 eV correspond to the signal associated with oxygen vacancies [92,93]. Notably, in the case of HBHP, the location of the deconvoluted O 1s peaks manifested evident shifts in comparison to their positions in pure Ho_2_YSbO_7_ and Bi_2_MoO_6_ samples. These shifts provided further evidence of interfacial interactions between Ho_2_YSbO_7_ and Bi_2_MoO_6_ species. Moreover, the spin–orbit disassociation numerical value between Sb 3d_5/2_ and Sb 3d_3/2_ was consistently measured as 8.03 eV for both Ho_2_YSbO_7_ and HBHP, which confirms the exclusive presence of Sb^5+^ species [94].

Furthermore, Figure S5 presents the XPS survey spectrum of NTO. The spectrum revealed the presence of O, Ti, C, and N elements, indicating the successful incorporation of nitrogen in the TiO_2_ [67,68,69]. These results, in conjunction with the XRD analysis, were consistent with previous findings and provide further evidence of the successful synthesis of NTO [67,68,69]. Moreover, Figure S6a,b display the spectral peaks corresponding to Ti 2p_3/2_, Ti 2p_1/2_, and N 1s, respectively, while Figure S6c exhibits the deconvoluted O 1s spectrum of NTO [67,68,69]. The presence of peaks at 531.41 eV, 530.21 eV, and 529.91 eV could be attributed to oxygen vacancies, hydroxyl groups, and lattice oxygen, respectively [92,93].

Based on the XPS analysis, it has been determined that the oxidation states of Ho, Y, Sb, Bi, Mo, and O ions in the material are +3, +3, +5, +3, +6, and −2, respectively. This finding confirms the successful fabrication of the photocatalytic samples based on the ideational chemical formula. Moreover, the surface elemental analysis revealed an average atomic ratio of Ho/Y/Sb/Bi/Mo/O as 826:411:407:854:419:7083, indicating the atomic ratios of Ho/Y/Sb and Bi/Mo in the HBHP sample of 2.03:1.01:1.00 and 2.04:1.00, respectively. No additional phases were observed by analyzing the XPS peaks of Ho_2_YSbO_7_ and Bi_2_MoO_6_, confirming their absence.

### 2.6. TEM-EDS Analysis

**Figure 7:**
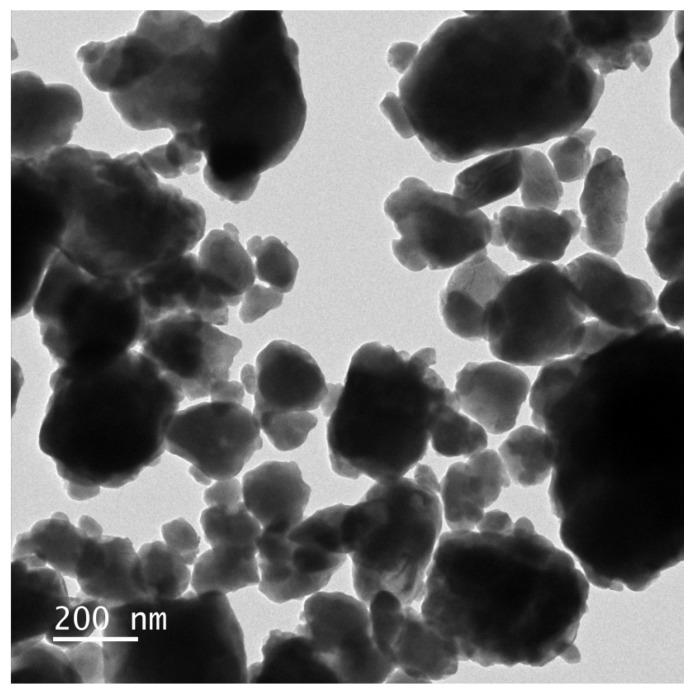
TEM image of HBHP.

**Figure 8:**
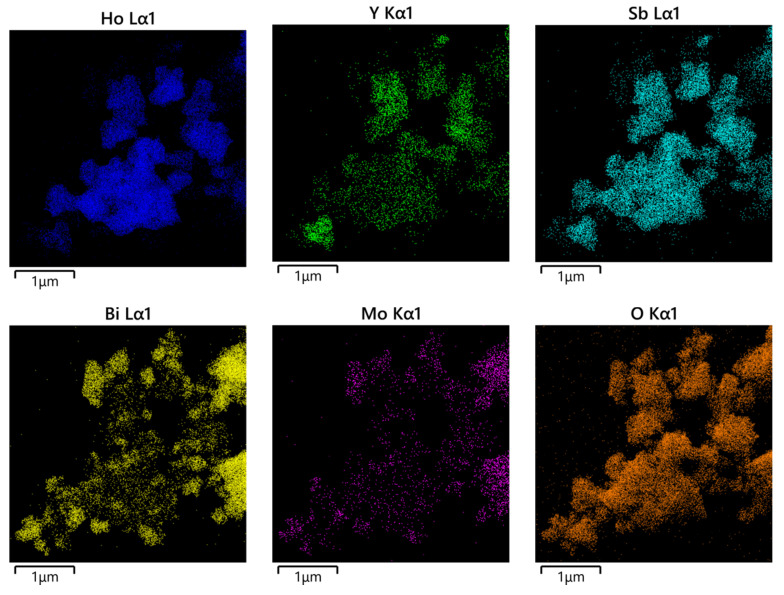
EDS elemental mapping of HBHP (Ho, Y, Sb, and O from Ho_2_YSbO_7_ and Bi, Mo, and O from Bi_2_MoO_6_).

The morphology and elemental composition of HBHP were investigated using transmission electron microscopy (TEM) and energy-dispersive X-ray spectroscopy (EDS). Figure 7 and Figure 8 depict TEM images and EDS elemental mapping of HBHP, respectively, with Ho, Y, Sb, and O derived from Ho_2_YSbO_7_, and Bi, Mo, and O from Bi_2_MoO_6_. The EDS spectrum of HBHP is presented in Figure S7. The EDS element mapping analysis presented in Figure 8 provided compelling evidence for the presence of Ho, Y, Sb, Bi, Mo, and O elements within the HBHP, thereby supporting the coexistence of Ho_2_YSbO_7_ and Bi_2_MoO_6_. Furthermore, through a careful examination of the light-emitting zones associated with Ho, Y, and Sb in comparison to Bi and Mo, it could be deduced that the larger microboulders correspond to Ho_2_YSbO_7_, while the smaller microboulders correspond to Bi_2_MoO_6_. This observation was substantiated by the visual arrangement of larger Ho_2_YSbO_7_ particles enveloped by the smaller Bi_2_MoO_6_ microparticles, as evident in Figure 7 and Figure 8. Consequently, these findings served as robust evidence validating the successful fabrication of the HBHP. Meanwhile, these findings were consistent with the XPS results depicted in Figure S4 and Figure 6. Additionally, the EDS spectrum (Figure S7) revealed an atomic ratio of approximately 826:413:409:853:421:7078 for Ho/Y/Sb/Bi/Mo/O, which closely matched the atomic ratio obtained from the XPS analysis. Based on these comprehensive results, it could be convincingly concluded that HBHP was successfully fabricated with high purity under the employed preparation conditions.

### 2.7. Photocatalytic Activity

#### Photocatalytic Activity in Photodegradation Experiments

The wastewater sample was collected from the Songhua River in Jilin Province, China. After pretreatment, the concentration of DRN in the wastewater was determined to be 0.032 mmol/L. The aim of this investigation was to assess the concentration variability profiles of DRN during the photodegradation process under VLE using a range of photocatalysts, including HBHP, Ho_2_YSbO_7_, Bi_2_MoO_6_, and NTO. NTO, a widely recognized visible-light-responsive photocatalyst, was employed as a benchmark to assess and compare the differences in photodegradation efficiency among various catalyst samples. As illustrated in Figure S8a, the experimental results clearly demonstrated a continuous decrease in DRN concentration during prolonged irradiation in the presence of the photocatalyst samples. This observation highlighted the effective degradation capability of the photocatalysts. In contrast, the photolysis assay results revealed no significant change in DRN concentration over the same extended period of irradiation in the absence of photocatalyst samples, confirming that the observed degradation could be attributed to the photocatalytic activity rather than mere photolysis.

To assess the REF of DRN, the equation ($1-\frac{C}{C_{0}}$) × 100% was utilized, with *C* representing the instantaneous saturation of DRN and *C*_0_ representing the initial saturation of DRN. Upon analysis of the data from Figure S8a, it was observed that the use of HBHP as the photocatalyst resulted in a remarkable DRN REF of 99.78% within pesticide wastewater after 152 min of VLE, corresponding to a reaction velocity of 3.50 × 10^−9^ mol·L^−1^·s^−1^ and a photonic efficiency (PEC) of 0.0735%. Similarly, when Ho_2_YSbO_7_ was employed as the photocatalyst, it showcased a conspicuous DRN REF of 88.44%, with a reaction velocity of 3.10 × 10^−9^ mol·L^−1^·s^−1^ and a PEC of 0.0651%. Furthermore, the use of Bi_2_MoO_6_ as the photocatalyst led to a pronounced DRN REF of 82.19%, accompanied by a reaction velocity of 2.88 × 10^−9^ mol·L^−1^·s^−1^ and a PEC of 0.0605%. In comparison, NTO exhibited a DRN REF of 33.78%, with a reaction velocity of 1.18 × 10^−9^ mol·L^−1^·s^−1^ and a photonic efficiency of 0.0248% [95,96].

These findings clearly indicate that HBHP demonstrated the highest photodegradation efficiency for DRN among the various photocatalysts studied. Additionally, the photodegradation efficiencies achieved with Ho_2_YSbO_7_ and Bi_2_MoO_6_ as photocatalysts also surpassed that of NTO. Furthermore, a comparison of the DRN degradation velocities after 152 min of VLE revealed the superior performance of HBHP, surpassing that of Ho_2_YSbO_7_ by 1.13 times, Bi_2_MoO_6_ by 1.21 times, and NTO by 2.95 times.

Figure S8b presents the saturation variation profiles of total organic carbon (TOC) throughout the photodegradation procedure of DRN in pesticide wastewater under VLE using different photocatalysts, including HBHP, Ho_2_YSbO_7_, Bi_2_MoO_6_, and NTO. The efficiency of the removal of TOC could be calculated by ($1-\frac{TOC}{TOC_{0}}$) × 100%, where *TOC* represents the instantaneous saturation of total organic carbon and *TOC_0_* represents the initial saturation of total organic carbon. An analysis of Figure S8b revealed that following 152 min of VLE, the REF values of TOC within pesticide wastewater were 97.19%, 83.33%, 77.81%, and 28.68% when employing HBHP, Ho_2_YSbO_7_, Bi_2_MoO_6_, and NTO as photocatalysts, respectively, for the treatment of DRN [97,98]. Moreover, a comparison of the TOC mineralization effectiveness after 152 min of VLE revealed the superior performance of HBHP, surpassing that of Ho_2_YSbO_7_ by 1.17 times, Bi_2_MoO_6_ by 1.25 times, and NTO by 3.39 times.

In conclusion, the results unequivocally showcased HBHP’s superior performance in terms of the REF for both DRN and TOC during the degradation process, outperforming Ho_2_YsbO_7_, Bi_2_MoO_6_, and NTO. Notably, the REF of TOC when utilizing Ho_2_YSbO_7_ significantly exceeded that of Bi_2_MoO_6_ or NTO. These findings underscore HBHP’s exceptional capabilities in both the mineralization and degradation of DRN, positioning it as a highly effective photocatalyst, surpassing the performance of Ho_2_YSbO_7_, Bi_2_MoO_6_, and NTO.

The concentration fluctuation graphs of DRN and TOC during five consecutive degradation cycles utilizing HBHP as a photocatalyst under VLE are illustrated in Figure S9a,b respectively. Following 152 min of VLE, the REF values of DRN were recorded at 99.78%, 98.66%, 97.63%, 96.56%, and 95.66% across the five cycles. Similarly, the REF values of TOC were documented at 97.19%, 96.11%, 95.10%, 94.06%, and 93.13% over the same cycles. These observations underscore the exceptional stability of HBHP during the degradation process. Moreover, a marginal reduction of 4.12% in the DRN degradation rate and a minor decrease of 4.06% in the TOC REF were noted throughout five consecutive degradation cycles. These findings indicate the remarkable structural stability of HBHP and its potential for effective reuse as a photocatalyst.

**Figure 9:**
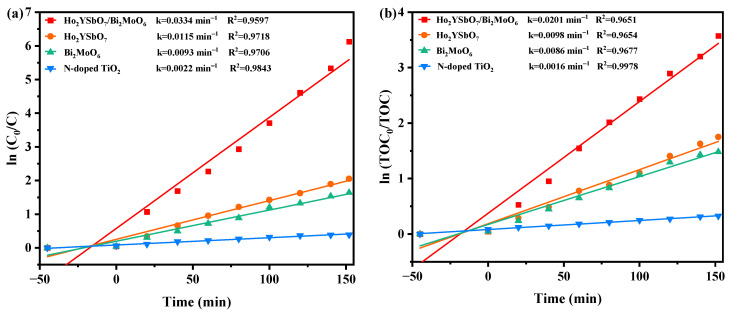
Observed first-order kinetics charts for (**a**) DRN and (**b**) TOC during photodegradation of DRN with HBHP, Ho_2_YSbO_7_, Bi_2_MoO_5_, or NTO as the catalytic sample under VLE.

Figure 9 presents the first-order kinetic plots of DRN (Figure 9a) and TOC (Figure 9b) during the photodegradation process of DRN using various photocatalysts (HBHP, Ho_2_YSbO_7_, Bi_2_MoO_6_, and NTO) under VLE. The kinetic constants were determined using the formulas ($ln\frac{C_{0}}{C}=k_{C}t)$ and ($ln\frac{{TOC}_{0}}{TOC}=k_{TOC}t)$, where *C*_0_ and *C* represent the initial and reaction saturation of DRN, and *TOC_0_* and *TOC* represent the initial and reaction saturation of total organic carbon. The calculated *k_C_* values from the dynamic DRN saturation versus light exposure time graphs were 0.0334 min^−1^, 0.0115 min^−1^, 0.0093 min^−1^, and 0.0022 min^−1^ for HBHP, Ho_2_YSbO_7_, Bi_2_MoO_6_, and NTO, respectively. Similarly, the *k_TOC_* values derived from the dynamic TOC saturation versus light exposure time curves were 0.0201 min^−1^, 0.0098 min^−1^, 0.0086 min^−1^, and 0.0016 min^−1^ for HBHP, Ho_2_YSbO_7_, Bi_2_MoO_6_, and NTO, respectively. Crucially, it is noteworthy that the *k_TOC_* values were consistently lower than the *k_C_* values for all the catalysts, indicating the generation of photodegradation intermediates during the process. Notably, HBHP exhibited significantly higher mineralization efficiency for DRN degradation compared to the other catalysts.

**Figure 10:**
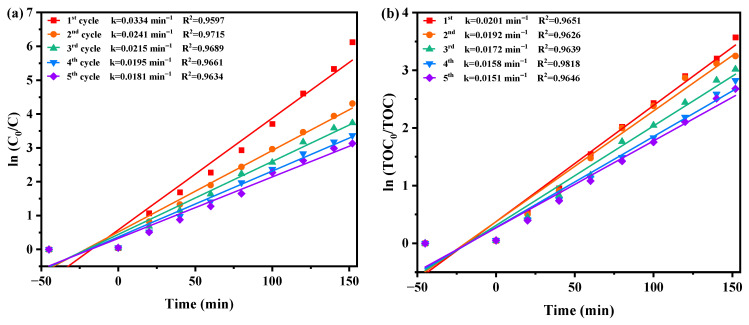
Observed first-order kinetic charts for (**a**) DRN and (**b**) TOC during photodegradation of DRN with HBHP as the catalytic sample under VLE for five cycle degradation tests.

Figure 10 displays the first-order kinetic plots of DRN and TOC during the photocatalytic degradation of DRN with HBHP, showcased in Figure 10a,b, respectively, under visible light conditions across five consecutive degradation cycles. The *k_C_* values derived from the dynamic DRN saturation versus light exposure time curves with HBHP as the sample for the five cycles in Figure 10a were determined as 0.0334 min^−1^, 0.0241 min^−1^, 0.0215 min^−1^, 0.0195 min^−1^, and 0.0181 min^−1^. Similarly, the *k_TOC_* values obtained from the dynamic TOC saturation versus light exposure time curves with HBHP as the sample for the three cycles were measured as 0.0201 min^−1^, 0.0192 min^−1^, 0.0172 min^−1^, 0.0158 min^−1^, and 0.0151 min^−1^. These findings validated the first-order reaction kinetics governing the photodegradation process of DRN in pesticide sewage using HBHP.

### 2.8. Property Characterization

**Figure 11:**
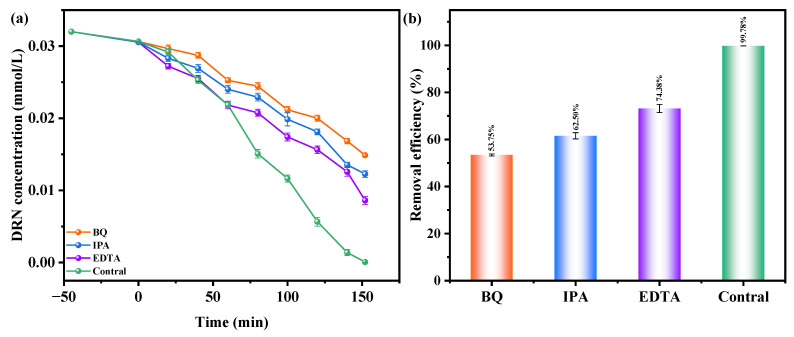
Effect of different radical scavengers on (**a**) DRN saturation and (**b**) removal efficiency of DRN.

Figure 11a,b investigate the influence of benzoquinone (BQ), isopropanol (IPA), and ethylene diamine tetraacetic acid (EDTA) as radical scavengers on the REF of DRN using HBHP as the catalytic material under VLE. IPA was used to apprehend •OH, BQ was employed to apprehend •O_2_^−^, and EDTA was utilized to apprehend h^+^. The scavengers were added during the initial stage of the photodegradation process to determine the active species responsible for degradation. Compared to the control group, the presence of BQ reduced the average DRN REF by 46.03%, while IPA and EDTA resulted in reductions of 37.28% and 29.78%, respectively. These results indicated the involvement of •O_2_^−^, •OH, and h^+^ as active radicals in the degradation process, with •O_2_^−^ exhibiting the highest oxidation removal capability. Thus, when HBHP acted as the photocatalyst, •O_2_^−^ radicals effectively eliminated DRN in pesticide-contaminated sewage. The ascending order of oxidation removal ability for degrading DRN was determined as •O_2_^−^ > •OH > h^+^.

**Figure 12:**
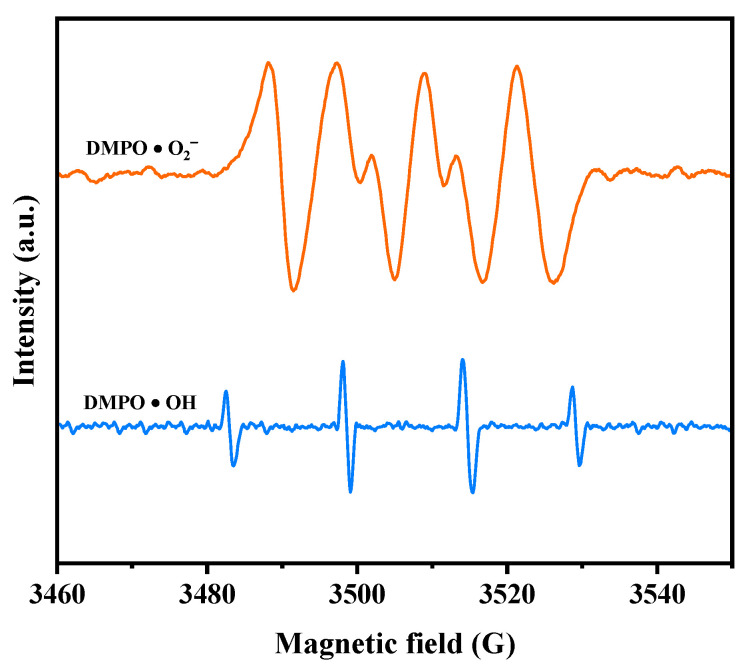
EPR spectrum for DMPO·O_2_^−^ and DMPO·OH over HBHP.

Electron paramagnetic resonance (EPR) analysis was conducted to investigate the generation of •O_2_^−^ radicals and •OH radicals during the photodegradation process. The EPR spectrum in Figure 12 illustrates the signals for DMPO•O_2_^−^ and DMPO•OH when HBHP was utilized as the photocatalyst. Following a 10 min exposure to visible light, the EPR spectrum exhibited a distinctive DMPO•O_2_^−^ signal with four strong peaks in an equal intensity ratio of 1:1:1:1, confirming the presence of •O_2_^−^ radicals [99]. Subsequently, the EPR spectrum displayed a four-line signal with a 1:2:2:1 intensity ratio, denoting the DMPO•OH signal acquired after VLE [99]. These observations implied the simultaneous generation of •O_2_^−^ radicals and •OH radicals during the photodegradation process. Notably, the relative intensity of the EPR signals indicates a higher yield of •O_2_^−^ radicals compared to •OH radicals [99,100]. These findings support the results obtained from the previous radical-scavenger experiments, further substantiating the involvement of •O_2_^−^ radicals and •OH radicals in the degradation mechanism.

To investigate the interfacial carriers’ optical properties, the dynamical behavior of the electronic excited states, and the lifetime, multiple measurement methodologies were employed. Figure S10a–d display the photoluminescence (PL) spectrum and time-Resolved photoluminescence (TRPL) spectra of Ho_2_YSbO_7_, Bi_2_MoO_6_, and HBHP. The PL spectrum was obtained using an excitation wavelength of 300 nm with a scan speed of 1200 nm/min. Higher PL intensities typically indicate faster recombination rates of the PIC, resulting in a lower photocatalytic effect [101,102]. In Figure S10a, HBHP exhibits the lowest radiative intensity, suggesting efficient charge separation and constrained recombination of the PIC. Ho_2_YSbO_7_ displayed a higher radiative intensity compared to HBHP, while Bi_2_MoO_6_ exhibited the highest radiative intensity among all three samples. These findings demonstrated that the heterostructure sample enhanced the photocatalytic efficiency towards DRN. Moreover, additional evidence was provided to support the superior photocatalytic performance of HBHP compared to Ho_2_YSbO_7_ and Bi_2_MoO_6_. The TRPL spectra in Figure S10b–d were fitted using a double-exponential decay equation (Equation (3)) [103]:

(3) $$I(t)=I_{0}+A_{1}exp(-\frac{t}{\tau_{1}})+A_{2}exp(-\frac{t}{\tau_{2}})$$

According to the presented equation, the first- and second-order decay times, *A*_1_ and *A*_2_, are weighting coefficients of each decay channel [104]. To determine the average PIC lifetime ($\tau_{ave}$), Equation (4) was utilized [105]:

(4) $$\tau_{ave}=(A_{1}\tau_{1}^{2}+A_{2}\tau_{2}^{2})/(A_{1}\tau_{1}+A_{2}\tau_{2})$$

The calculated lifetimes and corresponding parameters are presented in Table S1. HBHP exhibited significantly longer lifetimes ($\tau_{1}$ = 1.4139 ns, $\tau_{2}\;$= 130.9981 ns, $\tau_{ave}\;$= 99.7314 ns) compared to Ho_2_YSbO_7_ ($\tau_{1}\;$= 1.2890 ns, $\tau_{2}$= 116.1575 ns, $\tau_{ave}$ = 85.3321 ns) and Bi_2_MoO_6_ ($\tau_{1}\;$= 1.0946 ns, $\tau_{2\;}$= 11.6284 ns, $\tau_{ave}\;$= 5.7917 ns). These results unequivocally establish the unparalleled superiority of HBHP in terms of photocatalytic efficacy, surpassing that of the individual components Ho_2_YSbO_7_ and Bi_2_MoO_6_.

**Figure 13:**
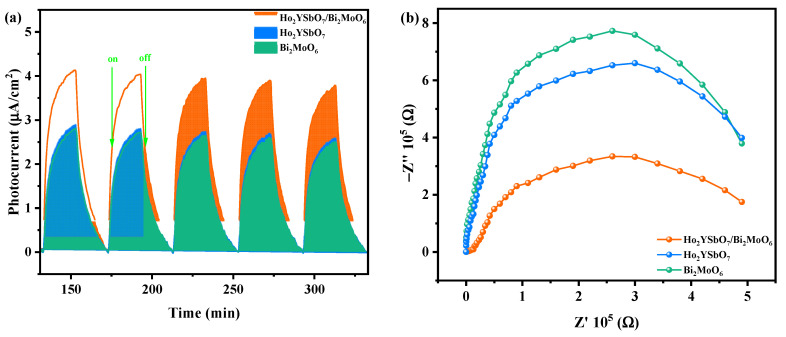
(**a**) Transient photocurrent and (**b**) Nyquist impedance plots of HBHP, Ho_2_YSbO_7_, and Bi_2_MoO_6_.

The influence of prepared HBHP, Ho_2_YSbO_7_, and Bi_2_MoO_6_ samples on the efficiency of electron–hole pair separation was investigated through photocurrent (PC) and electrochemical impedance spectroscopy (EIS) experiments. In Figure 13a, the transient photocurrent responses of HBHP, Ho_2_YSbO_7_, and Bi_2_MoO_6_ samples clearly demonstrated that HBHP exhibited the highest photocurrent response. This could be attributed to the efficient diffusion of photoexcited electrons, while the photoexcited holes rapidly transfer to the surface of Bi_2_MoO_6_ due to the electric potential difference of the valence band between Ho_2_YSbO_7_ and Bi_2_MoO_6_ within the HBHP [106]. The increased photocurrent response in HBHP indicated effective separation of the PIC and its prolonged lifetime during the photocatalytic degradation reaction [107]. Likewise, results from the EIS experiments in Figure 13b support this finding, with the relative arc sizes in the EIS Nyquist plot for the three electrodes following the order HBHP < Ho_2_YSbO_7_ < Bi_2_MoO_6_, confirming that HBHP exhibited the highest efficiency in charge separation [108,109]. The PC and EIS results underscored that the construction of a heterojunction significantly mitigated the recombination of electron–hole pairs and promoted the efficient separation of photogenerated charges compared with individual samples. These findings were consistent with the PL and TRPL analyses, further highlighting the exceptional photocatalytic efficacy achieved with HBHP.

### 2.9. Analysis of Possible Degradation Mechanisms 

**Figure 14:**
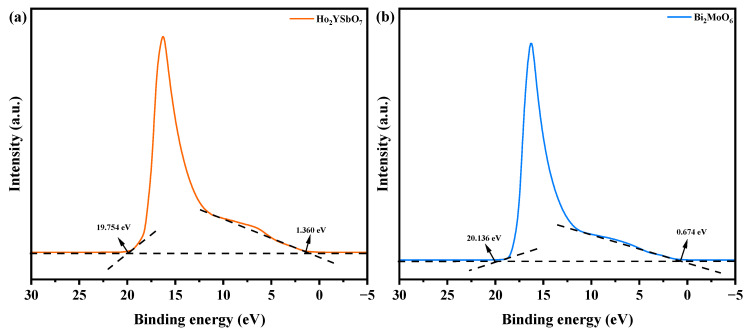
UPS spectra of (**a**) Ho_2_YSbO_7_ and (**b**) Bi_2_MoO_6_ (the intersections of the black dash lines indicated by the black arrows indicated the onset (Ei) and cutoff (Ecutoff) binding energy).

**Figure 15:**
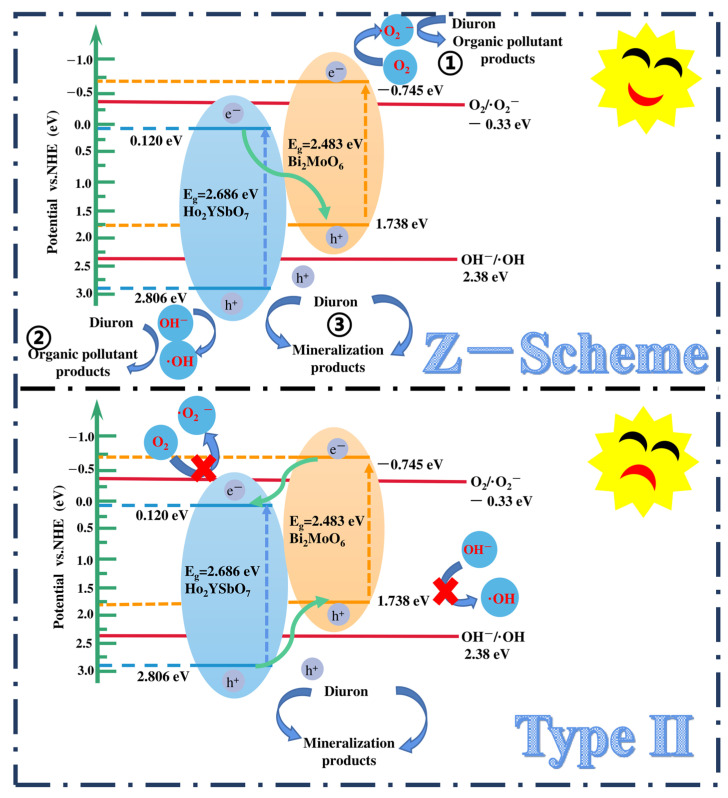
Possible photodegradation mechanism of DRN with HBHP as photocatalyst under VLE.

Figure 14 exhibits the UPS spectra of Ho_2_YSbO_7_ and Bi_2_MoO_6_, while Figure 15 illustrates the proposed photocatalytic degradation mechanism of DRN with HBHP as the catalytic sample under VLE. The UPS analysis aimed to precisely determine the ionization potential of Ho_2_YSbO_7_ and Bi_2_MoO_6_, with the onset (Ei) and cutoff (Ecutoff) binding energies for both samples shown in Figure 14 [110]. The measured values were 1.360 eV and 19.754 eV for Ho_2_YSbO_7_, and 0.674 eV and 20.136 eV for Bi_2_MoO_6_ [111]. Based on the excitation energy (approximately 21.2 eV), the ionization potential of Ho_2_YSbO_7_ and Bi_2_MoO_6_ was determined as 2.806 eV and 1.738 eV, respectively [112]. Consequently, the conduction band (CB) potential of Ho_2_YSbO_7_ and Bi_2_MoO_6_ was estimated as 0.120 eV and −0.745 eV, respectively. 

Based on the comprehensive and prominent analysis of the various heterojunctions proposed by Barrocas et al. [113] and the above analysis of the CB and VB positions of Ho_2_YSbO_7_ and Bi_2_MoO_6_, two potential photocatalytic mechanisms, namely the conventional type-II and direct Z-scheme heterojunction, could be considered to explain the enhanced transfer of PICs in this study, as illustrated in Figure 15. In the conventional type-II heterostructure, the photoexcited electrons and holes, generated at the CB (−0.745 eV) of Bi_2_MoO_6_ and VB (2.806 eV) of Ho_2_YSbO_7_, respectively, readily migrated to the CB (0.120 eV) of Ho_2_YSbO_7_ and the VB (1.738 eV) of Bi_2_MoO_6_. This migration was facilitated by the potential difference at the heterojunction. However, this migration resulted in a reduction in the redox potential of the photoinduced carriers. Moreover, the more positive CB edge of Ho_2_YSbO_7_ (0.120 eV) compared to the reduction potential of O_2_/•O_2_^−^ (−0.33 eV vs. NHE) [114] and the more negative VB edge of Bi_2_MoO_6_ relative to the oxidation potential of OH^−^/•OH (2.38 eV vs. NHE) made the formation of •O_2_^−^ and •OH species unfavorable [115,116]. Notably, these findings contradicted the results obtained from the active radical trapping experiment and EPR measurement, as demonstrated in Figure 11 and Figure 12. In light of these contradictory results, it was postulated that the direct Z-scheme heterojunction may provide a more appropriate explanation for the photocatalytic mechanism observed in the Ho_2_YSbO_7_/Bi_2_MoO_6_ heterojunction.

In the proposed Z-scheme mechanism in Figure 15, the photogenerated electrons were expected to migrate from the CB (0.120 eV) of Ho_2_YSbO_7_ to the VB (1.738 eV) of Bi_2_MoO_6_. This migration lead to the effective separation of PICs and the maintenance of higher reduction/oxidation potentials during photocatalytic reactions. Moreover, the electrons located in the CB (−0.745 eV) of Bi_2_MoO_6_ could react with O_2_, forming •O_2_^−^ species, which were responsible for the decomposition of DRN (① in Figure 15). Similarly, the electrons presented in the VB (2.806 eV) of Ho_2_YSbO_7_ could react with OH^−^, producing •OH species that participated in the decomposition of DRN (② in Figure 15). Furthermore, the photoinduced holes in the VB of Bi_2_MoO_6_ or Ho_2_YSbO_7_ could directly catalyze the oxidation and subsequent degradation of DRN due to its inherent strong oxidation capability (③ in Figure 15). Importantly, this analysis was consistent with the experimental results obtained through the active radical trapping experiment and EPR measurement, as demonstrated in Figure 11 and Figure 12. Therefore, it could be concluded that under VLE, the PIC migration pathway within the heterojunction composed of Ho_2_YSbO_7_ and Bi_2_MoO_6_ followed the direct Z-scheme. The significantly enhanced photocatalytic performance of the HBHP was closely associated with the successful construction of the direct Z-scheme heterojunction.

**Figure 16:**
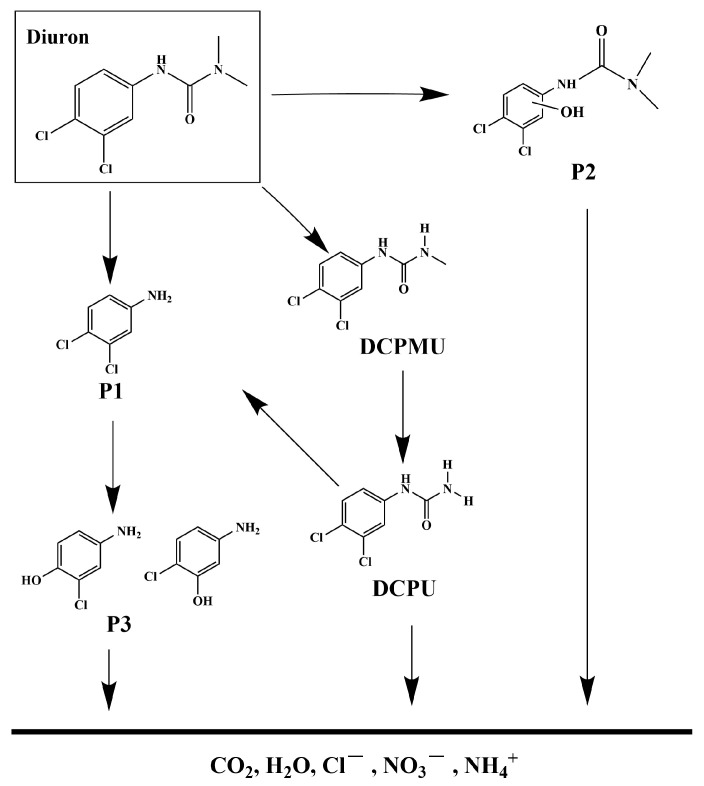
Suggested photodegradation pathway scheme for DRN under visible light condition with HBHP as photocatalytic sample.

The photocatalytic degradation system of DRN was investigated, and the intermediate products formed during the process were analyzed utilizing a liquid chromatograph–mass spectrometer (LC-MS). By referring to the previous literature, five main intermediates were identified, contributing to a comprehensive understanding of the degradation pathway of DRN [117,118]. A mechanistic scheme illustrating this pathway is depicted in Figure 16. Table S2 presents the identification of five intermediate products based on their respective molecular ions and mass fragment ions. The table also includes information such as the retention time (Rt), molecular weight (M.W.), and characteristic ions of these identified products. The degradation process commenced with •OH attacking specific sites on diuron, namely the methyl groups and the aromatic ring. Notably, this initial step did not involve dechlorination or alkyl chain modifications but led to the formation of four primary photoproducts: P1 (C_5_H_5_Cl_2_N, *m*/*z* = 162) [117], P2 (C_8_H_11_Cl_2_N_2_O_2_, *m*/*z* = 248) [117], 1-(3,4-dichlorophenyl)-3-methylurea (DCPMU, C_8_H_8_Cl_2_N_2_O, *m*/*z* = 219) [117], and 3,4-dichlorophenylurea (DCPU, C_7_H_6_Cl_2_N_2_O, *m*/*z* = 205) [118,119]. Subsequently, oxidation processes ensued, resulting in the elimination of alkyl groups and chlorine atoms. These reactions ultimately yielded the photoproduct P3 (C_6_H_6_ClNO, *m*/*z* = 143) [117,118]. In addition to these five compounds, other degradation products could still possibly exist in the photocatalytic system but were not detected because of their low concentration, extraction efficiency, and limited sensitivity in LC/MS. Many researchers have identified other degradation products during photocatalytic processes by HPLC, 1H NMR, and GC/MS, as well as these five products [117,119,120,121]. Finally, the oxidative opening of the aromatic ring occurred, generating the final products: Cl^−^, NO_3_^−^, NH_4_^+^, CO_2_, and H_2_O.

## 3. Experimental Section

### 3.1. Materials and Reagents

Ho(NO_3_)_3_·5H_2_O (purity = 99.99%), Y(NO_3_)_3_·6H_2_O (purity = 99.9%), and SbCl_5_ (purity = 99.999%) were procured from Shanghai Macklin Biochemical Co., Ltd. (Shanghai, China). Bi(NO_3_)_3_·5H_2_O (purity = 99.99%) and Na_2_MoO_4_ (purity = 99.9%) were obtained from Aladdin Group Chemical Reagent Co., Ltd. (Shanghai, China). Additionally, ethylenediaminetetraacetic acid (EDTA, C_10_H_16_N_2_O_8_, purity = 99.99%) and P-benzoquinone (BQ, C_6_H_4_O_2_, purity ≥ 99.0%) were acquired from Shanghai Macklin Biochemical Co., Ltd. (Shanghai, China). Isopropyl alcohol (IPA, C_3_H_8_O, purity ≥ 99.999%) was purchased from Aladdin Group Chemical Reagent Co., Ltd. (Shanghai, China). Furthermore, pure ethanol (C_2_H_5_OH, purity ≥ 99.5%) and DRN (C_9_H_10_Cl_2_N_2_O, purity ≥ 99.5%) were carefully selected and deemed suitable for gas chromatography, meeting the specifications set by the American Chemical Society. Both of the chemicals were acquired from Shanghai Macklin Biochemical Co., Ltd. (Shanghai, China).

### 3.2. Preparation Method of Bi_2_MoO_6_

The Bi_2_MoO_6_ catalyst was prepared via the solvothermal method in this study. First, equal volumes of Bi(NO_3_)_3_·5H_2_O (0.30 mol/L) and Na_2_MoO_4_ (0.15 mol/L) precursor solutions were meticulously mixed and stirred for 1200 min. Subsequently, the resulting precursor mixture was transferred into a high-pressure autoclave equipped with a polytetrafluoroethylene (PTFE) liner, followed by maintaining it at a temperature of 210 °C for 900 min. Next, the mixture was subjected to a controlled heating process in a tube furnace under a nitrogen (N_2_) atmosphere with a heating rate of 7.0 °C/min until it reached 750 °C. The mixture was then held at this temperature for 480 min. Consequently, pure Bi_2_MoO_6_ powder was successfully synthesized.

### 3.3. Preparation Method of Ho_2_YSbO_7_

The Ho_2_YSbO_7_ catalyst was prepared via the solvothermal method in this study. Firstly, equal volumes of Ho(NO_3_)_3_·5H_2_O (0.30 mol/L), Y(NO_3_)_3_·6H_2_O (0.15 mol/L), and SbCl_5_ (0.15 mol/L) precursor solutions were thoroughly mixed and stirred for a duration of 1500 min. Subsequently, the precursor mixture was transferred into a high-pressure autoclave equipped with a PTFE liner and maintained at a temperature of 220 °C for 800 min. Next, the resulting mixture was heated under a N_2_ atmosphere utilizing a tube furnace at a heating rate of 7.5 °C/min until reaching a temperature of 800 °C, and held at this temperature for 500 min. Finally, pure Ho_2_YSbO_7_ powder was successfully synthesized.

### 3.4. Synthesis of N-Doped TiO_2_

The sol–gel synthesis process was employed to fabricate NTO. Initially, a certain amount of butyl titanate was mixed with anhydrous ethanol to prepare solution M. Concurrently, glacial acetic acid, di-distilled water, and anhydrous ethanol were mixed to form solution N. Subsequently, solution M was added dropwise to solution N under magnetic stirring. After the complete addition, the mixture was stirred for an additional 30 min, resulting in a translucent gel-like suspension. Next, nitrogen doping was conducted by immersing the gel in urea to achieve a N/Ti mole ratio of 8 mol%. The suspension was stirred for an additional 60 min. Subsequently, the gel was air-dried at room temperature for 40 h, leading to the formation of a solid gel. The dried gel was crushed and subjected to calcination at 500 °C for 3.5 h. Finally, the NTO catalyst was obtained using a vibrating screen and utilized for subsequent investigation.

### 3.5. Synthesis of Ho_2_YSbO_7_/Bi_2_MoO_6_ Heterojunction Photocatalyst

HBHP was prepared by the hydrothermal method. First, equal volumes of Ho(NO_3_)_3_·5H_2_O (3 mol/L), Y(NO_3_)_3_·6H_2_O (1.5 mol/L), and SbCl_5_ (1.5 mol/L) were thoroughly mixed. The resulting solution was then transferred into a high-pressure reaction vessel lined with PTFE. The reaction medium consisted of a glycerol–water solution, with polyethylene glycol or ethylene glycol added as a dispersant. The solution volume occupied 60% of the high-pressure vessel. The high-pressure vessel was placed inside a high-temperature sintering furnace and heated to 240 °C while maintaining a pressure of 150 MPa for 1200 min. After the furnace cooled down to room temperature, the powder was extracted and subjected to centrifugal filtration. The resulting powder was washed three times with acetone, deionized water, and pure ethanol before being dried in a vacuum oven at 50 °C for 3 h. Subsequently, the dried powder mixture was compressed into thin slices and sintered in a high-temperature furnace. The heating process began at 20 °C and gradually increased to 950 °C over a duration of 100 min. The temperature was subsequently maintained at 950 °C for 600 min before cooling down with the furnace. Finally, pure Ho_2_YSbO_7_ powder was obtained.

Similarly, equal volumes of Bi(NO_3_)_3_·5H_2_O (3 mol/L) and Na_2_MoO_4_ (1.5 mol/L) were thoroughly mixed. The resulting solution was transferred into a high-pressure reaction vessel lined with PTFE, using the same reaction medium and dispersant as mentioned before. The volume of the solution occupied 60% of the high-pressure vessel. The high-pressure vessel was placed inside a high-temperature sintering furnace and heated to 200 °C while maintaining a pressure of 90 MPa for 1080 min. After cooling to room temperature, the powder was extracted and subjected to centrifugal filtration. The resulting powder was washed three times with acetone, deionized water, and pure ethanol before being dried in a vacuum oven at 50 °C for 3 h. Subsequently, the dried powder mixture was pressed into thin pellets and sintered in a high-temperature furnace. The heating process began at 20 °C and gradually increased to 850 °C over a duration of 90 min. The temperature was then maintained at 850 °C for 400 min before cooling down with the furnace. The sintered pellets were crushed into powder, resulting in the successful synthesis of pure Bi_2_MoO_6_.

Finally, a mixture of 1 mol of Ho_2_YSbO_7_ and 1 mol of Bi_2_MoO_6_ in 300 mL of octanol (C_8_H_18_O) was subjected to 60 min of ultrasonic treatment in an ultrasonic bath. The mixture was then rapidly stirred under reflux conditions at 150 °C for 150 min to facilitate the adhesion of Bi_2_MoO_6_ onto the surface of Ho_2_YSbO_7_ nanoparticles, forming Ho_2_YSbO_7_/Bi_2_MoO_6_ heterojunction photocatalytic materials. After cooling to room temperature, the product was obtained through centrifugation and washed three times with a mixture of n-hexane and ethanol. The purified powder was dried in a vacuum oven at 50 °C for 5 h and stored in a desiccator for further use. Ultimately, Ho_2_YSbO_7_/Bi_2_MoO_6_ heterojunction photocatalytic materials were successfully fabricated.

### 3.6. Characterization

The pure-phase Ho_2_YSbO_7_ and Bi_2_MoO_6_ samples, synthesized via the hydrothermal synthesis technique, were characterized using a comprehensive array of advanced analytical techniques. These include X-ray diffractometry (XRD), UV-Vis diffuse reflectance spectrophotometry (UV-Vis DRS), Fourier transform infrared spectroscopy (FTIR), Raman spectroscopy, X-ray photoelectron spectroscopy (XPS), transmission electron microscopy (TEM), energy dispersive X-ray spectroscopy (EDS), ultraviolet photoelectron spectroscopy (UPS), a fluorescence spectrophotometer, and electron paramagnetic resonance (EPR) spectroscopy. XRD analysis was performed using an X-ray diffractometer (XRD-6000, Shimadzu Corporation, Kyoto, Japan) to obtain crystallographic information. Microstructural and morphological features were examined using transmission electron microscopy (TEM, JEM—F200 FEI Tecnai G2 F20 FEI Talos F200s), and the elemental composition was determined through energy-dispersive spectroscopy (EDS). UV-Vis diffuse reflectance spectrophotometry (UV-Vis DRS, UV-3600, Shimadzu Corporation, Kyoto, Japan) was employed to study the optical properties of the samples. Functional groups and chemical bonds were analyzed using a Fourier infrared spectrometer (FTIR, WQF-530A, Beifen-Ruili Analytical Instrument (Group) Co., Ltd., Beijing, China). Raman spectrometry (INVIA0919-06, RENISHAW plx, Wotton-under-Edge, Gloucestershire, GL12 8JR, London, UK) was employed to investigate chemical bond interactions. The surface chemical composition and states were examined using X-ray photoelectron spectroscopy (XPS, PHI 5000 VersaProbe, UlVAC-PHI, Maoqi City, Japan). Ultraviolet photoelectron spectroscopy (UPS, Escalab 250 xi, Thermo Fisher Scientific, Waltham, MA, USA) was utilized to measure the ionization potential of the valence band. The related information about the free radicals in the samples was detected using an electron paramagnetic resonance spectrometer (EPR, A300, Bruker Corporation, Karlsruhe, Germany). The properties of PICs were determined using a fluorescence spectrophotometer (FLS980, Edinburgh Instruments Ltd., Edinburgh, UK).

### 3.7. Photoelectrochemical Experiments

The electrochemical impedance spectroscopy (EIS) tests were performed using a CHI660D electrochemical workstation (Chenhua Instruments Co., Ltd., Shanghai, China) with a standard three-electrode setup. The working electrode consisted of the as-fabricated materials, while a platinum plate served as the counter electrode. A Ag/AgCl electrode was used as the reference electrode. A 0.5 mol/L Na_2_SO_4_ aqueous solution was used as the electrolyte. During the experiment, light simulation was carried out using a 500 W Xe lamp equipped with a 420 nm cutoff filter.

### 3.8. Experimental Setup and Procedure

The degradation experiments were conducted using a photocatalytic reactor (CEL-LB70, China Education Au-Light Technology Co., Ltd., Beijing, China). The visible light condition was simulated by a 500 W xenon lamp and a 420 nm cutoff filter.

In each experiment, 12 quartz tubes were used and each quartz tube was filled with a reaction solution with a volume of 40 mL. For chemical industry wastewater, the total reaction volume was 480 mL. The dosage of nanophase materials (Ho_2_YSbO_7_, Bi_2_MoO_6_, or HBHP) in each experiment was maintained at 0.5 g/L. The initial concentration of DRN in the solution was 0.025 mmol/L.

During the reaction, 3 mL samples of the dispersed system were periodically collected for analysis. After 152 min of light exposure, a 13 mL sample of the dispersed system was collected to measure the residual DRN concentration. The photocatalyst was then removed by filtration using a 0.22 µm PES (polyether sulfone) filter membrane. The remaining DRN concentration was determined using Agilent 200 high-performance liquid chromatography (Agilent Technologies, Palo Alto, CA, USA). In the post-photodegradation process of the DRN dispersion system, a volume of 10 µL was injected at a flow rate of 1 mL/min.

To establish adsorption/desorption equilibrium between the photocatalytic sample, DRN, and atmospheric oxygen, the dispersed system containing the sample and DRN was stirred in darkness for 45 min prior to light exposure. During light exposure, the dispersed system was stirred at a speed of 600 rpm.

The mineralization of DRN in the dispersed system was assessed using a TOC analyzer (TOC-5000 A, Shimadzu Corporation, Kyoto, Japan). In order to calibrate the TOC saturation during the DRN photocatalytic process, either potassium acid phthalate (KHC_8_H_4_O_4_) or anhydrous sodium carbonate was utilized as the reference reactant. Calibration standards were prepared by using potassium acid phthalate with known carbon concentrations ranging from 0 to 100 mg/L. TOC saturation was evaluated across six samples, with each containing a reaction solution volume of 40 mL.

For the calibration experiments of intermediate reactants, liquid chromatography–mass spectrometry (LC-MS, Thermo Quest LCQ Duo, Thermo Fisher Scientific Corporation, Waltham, MA, USA) was employed. A Beta Basic-C18 HPLC column (150 mm × 2.1 mm, 5 µm ID, Thermo Fisher Scientific Corporation, Waltham, MA, USA) was used during the DRN photocatalytic process. After the photocatalytic reaction, a 20 µL solution resulting from the reaction was injected into the LC-MS system automatically. The system employed a mobile phase consisting of a mixture of 60% methanol and 40% ultrapure water. The mobile phase flowed through the system at a rate of 0.2 mL/min. The spray voltage was adjusted to 4800V, while maintaining the capillary temperature at 27 °C and the voltage at 19.00 V. For analysis, the mass-to-charge ratio (*m*/*z*) range was configured from 50 to 500.

In order to evaluate the photon intensity of the irradiating light, a 420 nm filter was utilized to select the desired range of radiation within the visible light spectrum. The calculation of the number of photons passing through the filter per unit time, whether total photons or reactive photons, could be performed using the formula υ = c/λ. In this equation, υ represents the frequency of photons, λ corresponds to the wavelength of incident light, and c denotes the speed of light. By utilizing the precise numerical values of the Avogadro constant (NA) and Planck constant (h), the energy of a photon (hν) could be accurately determined. The irradiation photon flux could be adjusted by varying the distance between the photoreactor and the light source. To measure the incident photon flux, *I_o_*, a radiometer (Model FZ-A, Photoelectric Instrument Factory Beijing Normal University, Beijing, China) was employed. The determined numerical value under light exposure was 4.76 × 10^−6^ Einstein L^−1^ s^−1^. The PEC was determined with Equation (5):

*ϕ* = *R*/*I_o_* (5)

where *ϕ* is the PEC (%), *R* is the retrogradation velocity of DRN (mol L^−1^ s^−1^), and *I_o_* is the irradiation photon flux (Einstein L^−1^ s^−1^). 

## 4. Conclusions

A groundbreaking Ho_2_YSbO_7_ photocatalyst was successfully synthesized using the solvothermal synthesis technique, while HBHP was synthesized through the hydrothermal fabrication technique. Characterization of the synthesized samples involved a comprehensive suite of techniques, including X-ray diffraction (XRD), ultraviolet–visible diffuse reflectance spectroscopy (UV-Vis DRS), Fourier transform infrared (FTIR) spectroscopy, Raman spectroscopy, X-ray photoelectron spectroscopy (XPS), transmission electron microscopy (TEM), energy-dispersive X-ray spectroscopy (EDS), a photocurrent (PC) test, electrochemical impedance spectroscopy (EIS), ultraviolet photoelectron spectroscopy (UPS), photoluminescence (PL) spectroscopy, and electron paramagnetic resonance (EPR) spectroscopy. The formation of the heterostructure between Ho_2_YSbO_7_ and Bi_2_MoO_6_ played a crucial role in enhancing the photocatalytic efficiency for pollutant degradation. Notably, HBHP exhibited outstanding removal performance for DRN in wastewater, achieving a remarkable REF of up to 99.78% for DRN and 97.19% for TOC within a short exposure time of 152 min. Comparative analysis demonstrated that HBHP displayed a significantly higher REF for DRN compared to the Ho_2_YSbO_7_, Bi_2_MoO_6_, and NTO photocatalysts, with the REF being 1.13 times, 1.25 times, and 2.95 times higher, respectively. The superior photocatalytic activity of HBHP could be attributed to its efficient separation and low recombination of PICs. The formation of the heterojunction between Ho_2_YSbO_7_ and Bi_2_MoO_6_ facilitated effective charge migration and the generation of free radicals, thus enhancing the degradation of DRN. This study identified •O_2_^−^ radicals as the primary reactive species responsible for the degradation of DRN, with •OH and h^+^ radicals also participating in the degradation process. Additionally, a plausible degradation pathway and mechanism for DRN were proposed. These findings offer promising strategies for the treatment of DRN-contaminated wastewater and are of great significance for the advancement of photocatalytic technology and the development of highly efficient nanophase photocatalytic materials for environmental pollutant degradation.

## Figures and Tables

**Table 1:** Structural eigenvalues of Ho_2_YSbO_7_ fabricated by solvothermal synthesis technique.

Atom	x	y	z	Occupation Factor
Ho	0	0	0	1
Y	0.5	0.5	0.5	0.5
Sb	0.5	0.5	0.5	0.5
O(1)	−0.173	0.125	0.125	1
O(2)	0.125	0.125	0.125	1

**Table 2:** Structural eigenvalues of Bi_2_MoO_6_ fabricated by solvothermal synthesis technique.

Atom	x	y	z	Occupation Factor
Bi(1)	0.517	0.426	0.983	0.383
Bi(2)	0.473	0.078	0.969	0.602
Mo	0.005	0.244	0.000	0.182
O(1)	0.057	0.140	0.076	0.850
O(2)	0.259	0.999	0.263	0.690
O(3)	0.240	0.500	0.255	0.400
O(4)	0.705	0.232	0.250	0.790
O(5)	0.213	0.263	0.330	0.960
O(6)	0.516	0.359	0.561	0.690

## Data Availability

Data are contained within the article.

## Supplementary Materials

The following supporting information can be downloaded at https://www.mdpi.com/article/10.3390/ijms25084418/s1.
