# Supplementary material for: The Fabrication and Property Characterization of a Ho2YSbO7/Bi2MoO6 Heterojunction Photocatalyst and the Application of the Photodegradation of Diuron under Visible Light Irradiation
Source: Int J Mol Sci. 2024 Apr 17;25(8):4418. doi: 10.3390/ijms25084418 (PMC11050021; doi:10.3390/ijms25084418)
Supplement: Supplementary file 1 [file ijms-25-04418-s001.zip › ijms-2944512-supplementary.pdf]

Fabrication, Property Characterization of Ho_2YSbO_7/Bi_2MoO_6 Heterojunction Photocatalyst and the Application of the Photodegradation of Diuron under Visible Light Irradiation

Liang Hao [1] and Jingfei Luan [1,2,*]

[1] School of Physics, Changchun Normal University, Changchun 130032, China; hliang0725@163.com (L.H.)
[2] State Key Laboratory of Pollution Control and Resource Reuse, School of the Environment, Nanjing University, Nanjing 210093, China
* Correspondence: jfluan@nju.edu.cn; Tel.: +86-19951939498

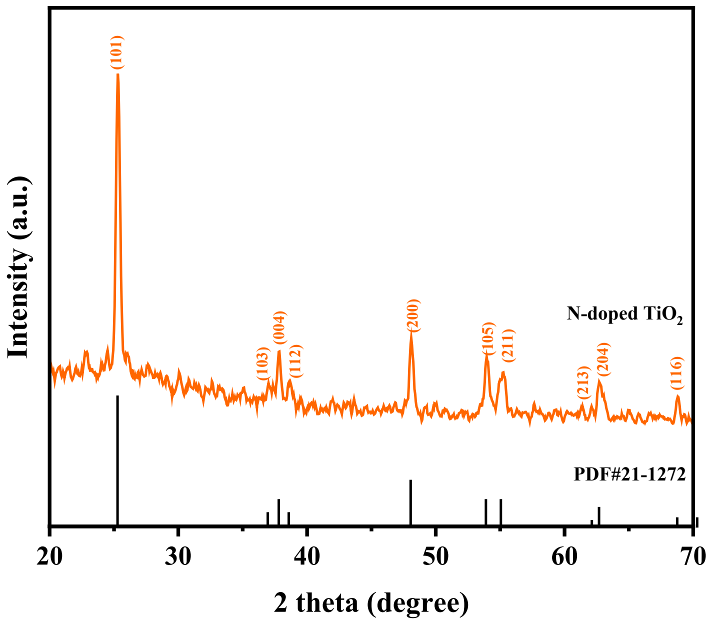

Figure S1. XRD imagery of N-doped TiO_2.

The calculation process of "n" value are shown as below: Following the equation (2), E_g and n could be determined: (1) plotting ln ($\alpha h\nu$) versus ln ($h\nu - E_g$) assuming an approximate value of E_g; (2) deducing the value of n in accordance with the slope in this graph; (3) refining the value of E_g by plotting $(\alpha h\nu)^{1/n}$ versus $h\nu$ and extrapolating the plot to $(\alpha h\nu)^{1/n}$ = 0. First, the direct method (1240/transition wavelength λ) was used to estimate the band gap E_g of Ho_2YSbO_7 or Bi_2MoO_6; as a result, the E_g of Ho_2YSbO_7 or Bi_2MoO_6 was estimated to be 2.750 eV (1240/450) or 2.455 eV (1240/505). Secondly, an indirect method (Equation (2)) was utilized to measure the precise band gap width of Ho_2YSbO_7 or Bi_2MoO_6 using 2.750 eV or 2.455 eV within Equation (2). Based on the above steps, the values of E_g for Ho_2YSbO_7 or Bi_2MoO_6 were calculated to be 2.686 eV or 2.483 eV. The value of n was determined to be approximately 2, indicating that the optical transition in Ho_2YSbO_7 or Bi_2MoO_6 was indirectly allowed, as shown in Figure S2.

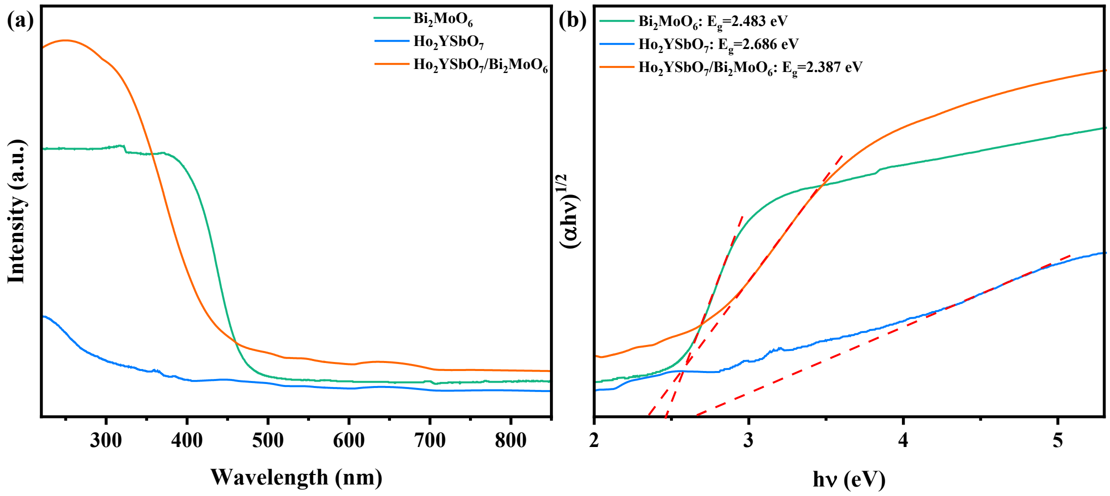

Figure S2. (a) The UV-Vis diffuse reflectance spectra and **(b)** Correlative diagram of $(\alpha h\nu)^{1/2}$ and $h\nu$ of the synthesized HBHP, Ho_2YSbO_7 and Bi_2MoO_6.

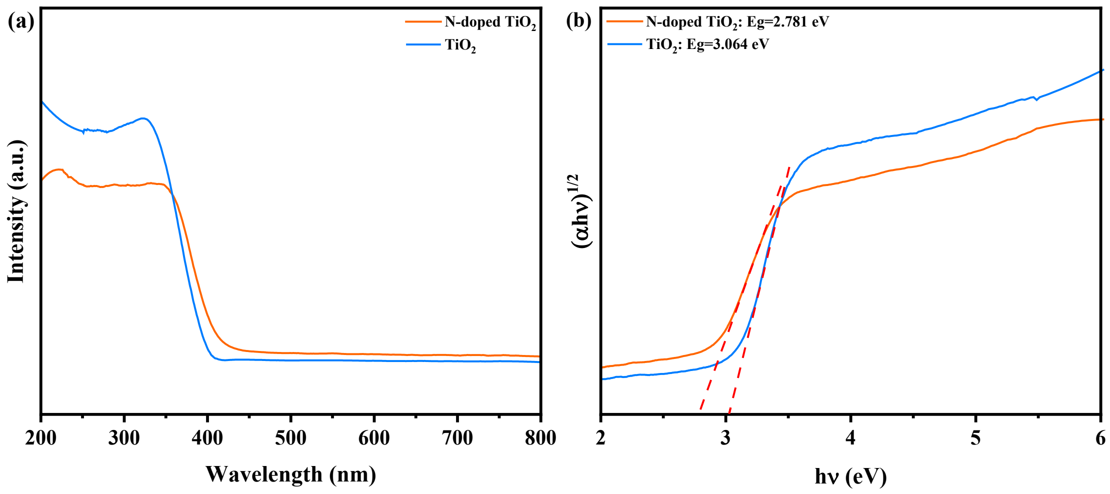

Figure S3. (a) The UV-Vis diffuse reflectance spectra and **(b)** Correlative diagram of $(\alpha h\nu)^{1/2}$ and $h\nu$ of N-doped TiO_2 and TiO_2.

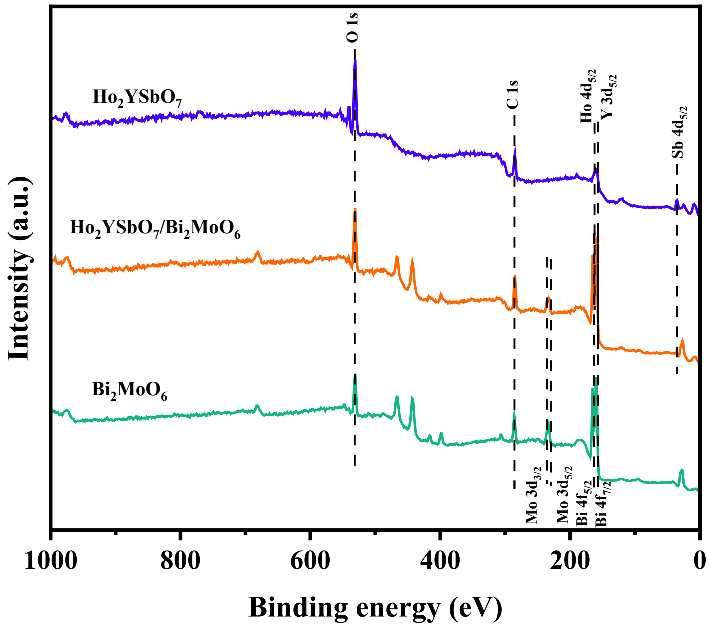

Figure S4. The XPS full spectrum of the synthesized HBHP, Ho_2YSbO_7 and Bi_2MoO_6.

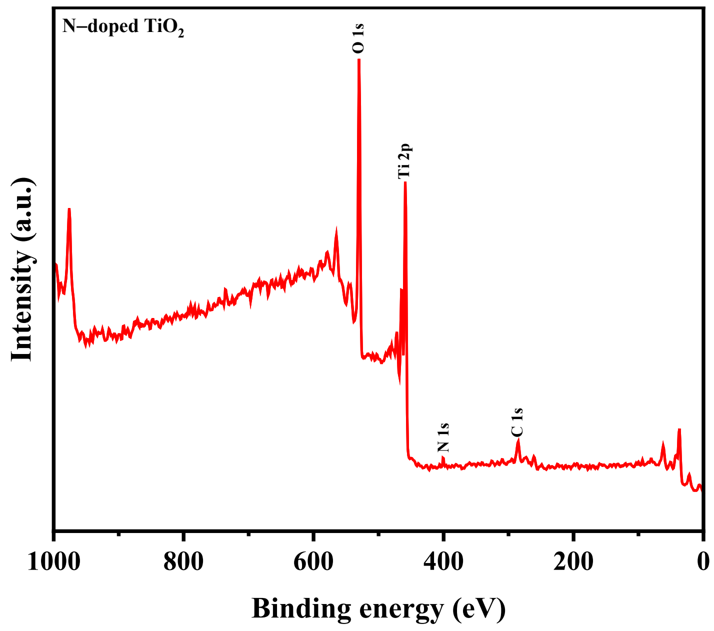

Figure S5. The XPS full spectrum of N-doped TiO_2.

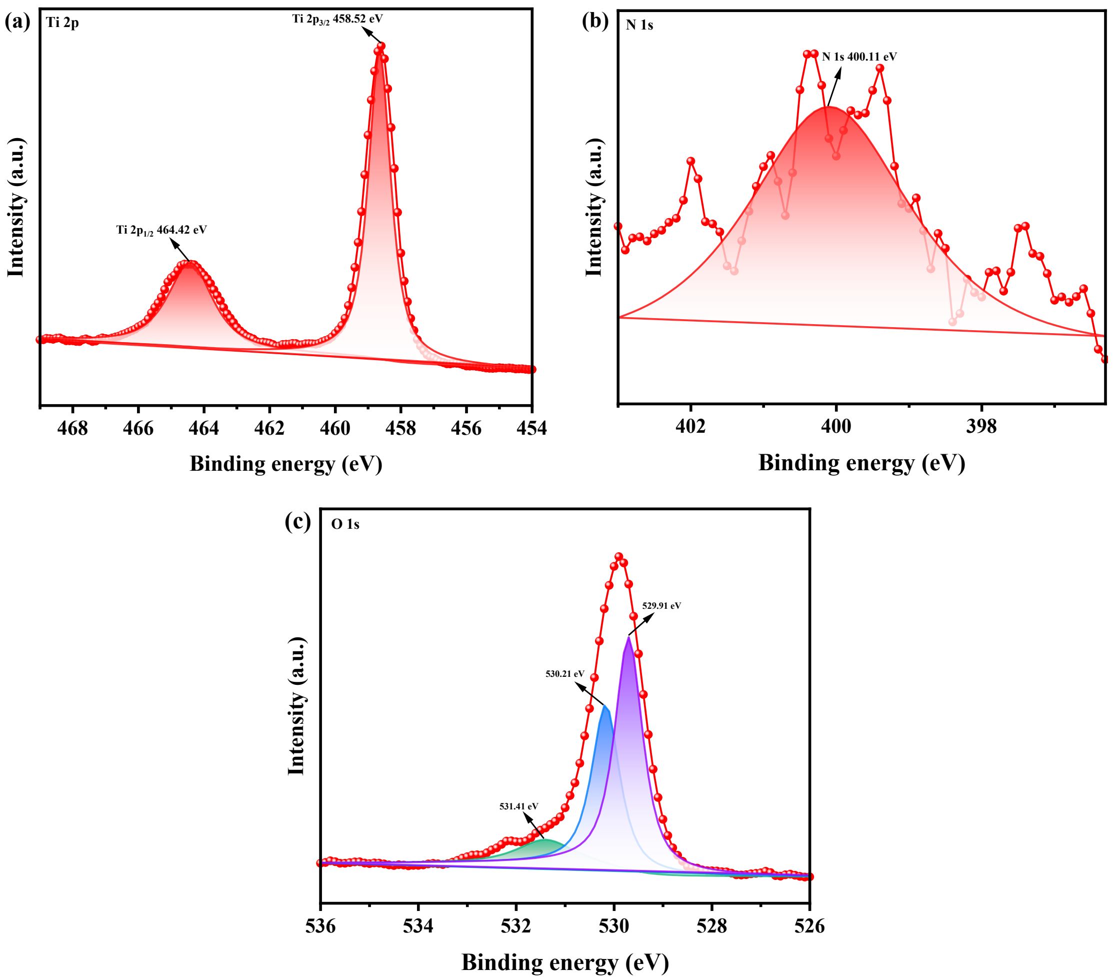

Figure S6. The corresponding high-resolution XPS spectra of **(a)** Ti 2p, **(b)** N 1s and **(c)** O 1s of N-doped TiO_2.

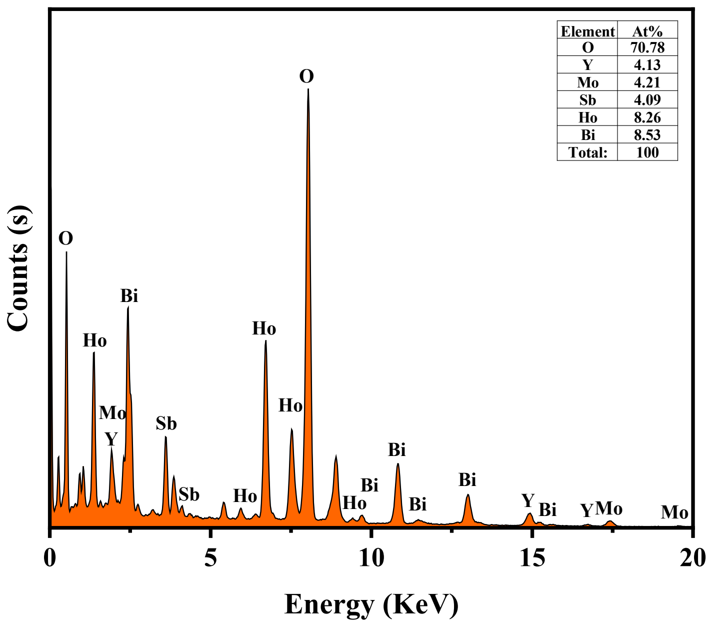

Figure S7. The EDS spectrum of HBHP.

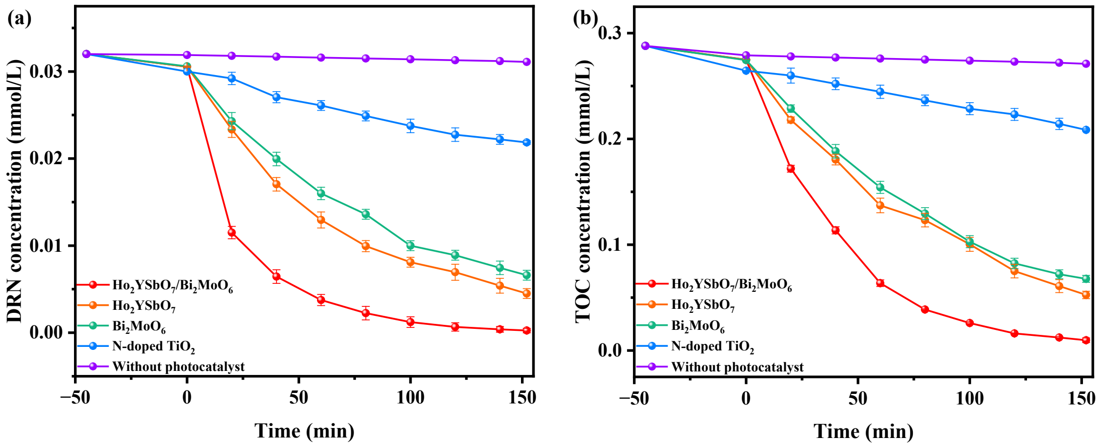

Figure S8. Concentration fluctuation graphs of **(a)** DRN and **(b)** TOC during photodegradation of DRN with HBHP, Ho_2YSbO_7, Bi_2MoO_6, NTO or without sample as the catalytic sample under VLE.

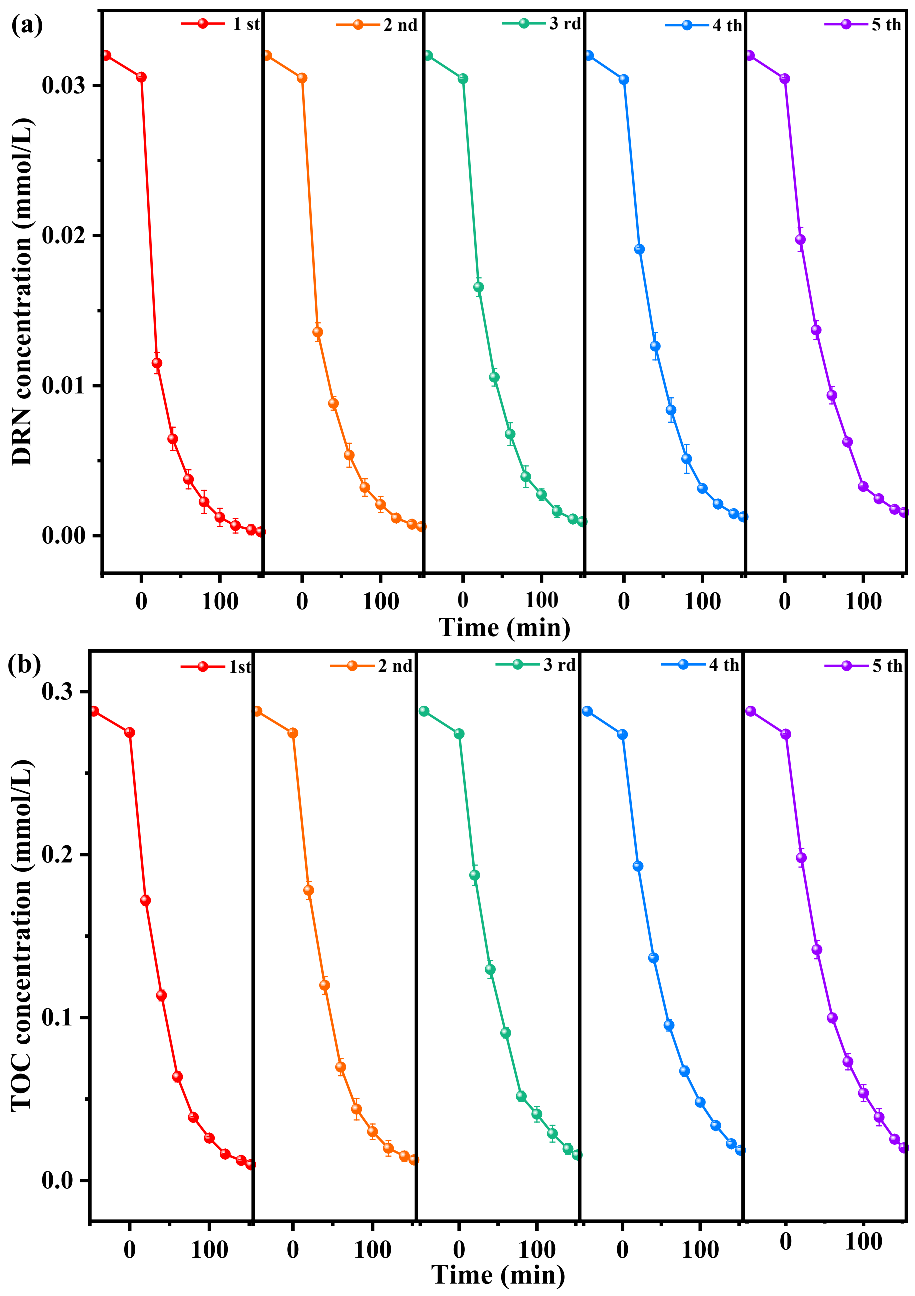

Figure S9. Concentration fluctuation graphs of **(a)** DRN and **(b)** TOC during photodegradation of DRN in pesticides wastewater with HBHP as photocatalyst under VLE for five consecutive degradation cycles tests.

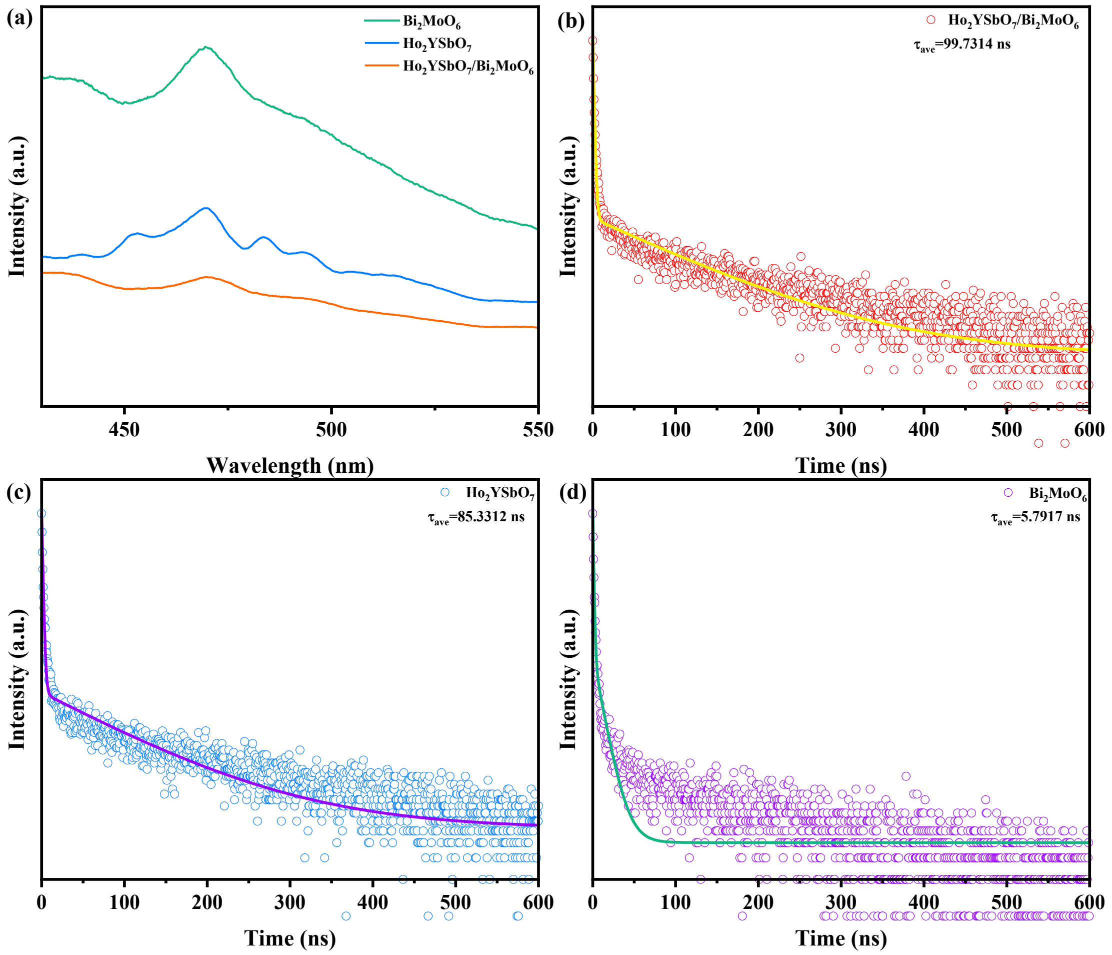

Figure S10. (a) PL spectrum of HBHP, Ho_2YSbO_7 and Bi_2MoO_6, and TRPL spectra of **(b)** HBHP, **(c)** Ho_2YSbO_7 and **(d)** Bi_2MoO_6.

Table S1. Fitted results of TRPL curves of Ho_2YSbO_7, Bi_2MoO_6 and HBHP.

	Ho_2YSbO_7	Bi_2MoO_6	Ho_2YSbO_7/Bi_2MoO_6
A_1	0.9438	0.9186	0.9547
τ_1 (ns)	1.289	1.0946	1.4193
A_2	0.0316	0.0676	0.0325
τ_2 (ns)	116.1575	11.6284	130.9981
τ_{ave} (ns)	85.3321	5.7917	99.7314

Table S2. The identification of degradation products by LC/MS during photocatalytic degradation of diuron.

Product	Rt (min)	M.W.	Characteristic ions (m/z)
Diuron	13.5	233	233, 177, 72
DCPMU	10.3	219	219, 167, 127
DCPU	10.1	205	205,167, 127
P1	11.3	162	162, 128, 105
P2	14.7	248	248, 207, 72
P3	6.5	143	143, 71, 126
